# Chromatographic and Spectroscopic Identification and Recognition of Natural Dyes, Uncommon Dyestuff Components, and Mordants: Case Study of a 16th Century Carpet with Chintamani Motifs

**DOI:** 10.3390/molecules23020339

**Published:** 2018-02-06

**Authors:** Olga Otłowska, Marek Ślebioda, Agata Kot-Wasik, Jakub Karczewski, Magdalena Śliwka-Kaszyńska

**Affiliations:** 1 Faculty of Chemistry, Gdansk University of Technology, 80-233 Gdansk, Poland; olgotlow@student.pg.edu.pl (O.O.); agawasik@pg.edu.pl (A.K.-W.); 2 Perlan Technologies, Sp. z.o.o., 02-785 Warszawa, Poland; m.slebioda@wp.pl; 3 Faculty of Applied Physics and Mathematics, Gdansk University of Technology, 80-233 Gdansk, Poland; jkarczew@mif.pg.gda.pl

**Keywords:** natural dyes, flavonoids, flavone glycosides, anthraquinones, extraction procedure, liquid chromatography mass spectrometry

## Abstract

A multi-tool analytical practice was used for the characterisation of a 16th century carpet manufactured in Cairo. A mild extraction method with hydrofluoric acid has been evaluated in order to isolate intact flavonoids and their glycosides, anthraquinones, tannins, and indigoids from fibre samples. High-performance liquid chromatography coupled to spectroscopic and mass spectrometric detectors was used for the identification of possible marker compounds with special attention paid to natural dyes present in the historical samples. Weld, young fustic, and soluble redwood dye were identified as the dye sources in yellow thread samples. Based on the developed method, it was possible to establish that red fibres were coloured with lac dye, whereas green fibre shades were obtained with indigo and weld. Tannin-containing plant material in combination with indigo and weld were used to obtain the brown hue of the thread. Hyphenation of high-performance liquid chromatography (HPLC) with quadrupole time-of-flight mass spectrometry (QTOF MS) and triple-quadrupole mass spectrometry (QqQ MS) enabled us to recognise four uncommon and thus-far unknown dye components that were also found in the historical samples. These compounds probably represent a unique fingerprint of dyed threads manufactured in a Turkish workshop. Scanning electron microscopy with energy-dispersive X-ray detector (SEM-EDS) and Fourier transform infrared spectroscopy (FT-IR) were used for the identification and characterisation of substrates and mordants present in the historical carpet. Carbon and oxygen were detected in large quantities as a part of the wool protein. The presence of aluminium, iron, and calcium indicated their usage as mordants. Trace amounts of copper, silica, and magnesium might originate from the contaminants. FT-IR analysis showed bands characteristic for woollen fibres and SEM micrographs defined the structure of the wool.

## 1. Introduction

Scientific analysis of objects of artistic and historic significance is the key to reconstructing their story and elucidating the circumstances under which they have been created. Investigation of the origin, nature, and chemical behaviour of the coloured materials used in the production of historical artefacts may shed new light on their original colour and appearance [1]. Knowledge of historical artworks’ components essential for documentation of their authenticity requires major breakthroughs in interdisciplinary collaborations between archaeologists and analytical chemists [2]. For this reason, many analytical techniques have been employed in the investigation of natural dyestuffs. Identification of colorants in artworks and objects of historical value poses a whole set of analytical challenges due to the wide range of possible dye sources and the vast number of chemical classes they belong to, the small amount of sample available for analysis, and the low amount of coloured compounds often present at trace levels [3]. The first objective of historical textile reconstruction is the identification of fibres’ constitution. They are generally made of organic materials, such as plant and/or animal fibres or silk. Fourier transform infrared spectroscopy (FT-IR) and Scanning electron microscopy with energy-dispersive X-ray detector (SEM-EDS) are powerful tools for fibres’ characterisation and enable us to distinguish their origin and identify mordants [4,5,6]. Until the mid-19th century, the only sources of dyes for textiles and other fibres were natural materials of vegetal or animal origin. In general, all shades of colour were made from combinations of the primary colours: blue, red, and yellow. An exact identification of the colouring substances in biological sources gives us the information necessary to determine the origin of dyestuffs used to create the object. This data, with additional information about metal ions (mordant-type dyes) and the type of textile raw material used to produce an artefact, can help in determining how, when, and where these works of art were made.

One approach to achieve high sensitivity of the analytical procedure is to improve the dye extraction process. It is crucial to fully extract the information that can lead to the identification of a particular plant or other dyestuff used to colour a textile. Since most textiles are mordanted with metal ions, the extracting solution must be able to disrupt the dye–metal complex. The conventional method of a dye’s isolation from textile fibres is usually carried out with boiling a hydrochloric acid–methanol mixture. This procedure allows for the efficient isolation of flavonoids and anthraquinone dyestuffs from textile fibres but causes hydrolysis of their glycosidic forms to parent aglycones [7,8]. Moreover, the use of hydrochloric acid is ineffective for the extraction of indigotin and indirubin [9]. Blue indigoid dyes are better extracted with hot pyridine, pyridine with water [10,11], dimethylformamide, or dimethylsulfoxide [12,13]. For this reason, milder extraction methods based on the use of ethylenediaminetetraacetic acid (EDTA) or citric, tartaric, formic, or hydrofluoric acid are currently being tested [14,15,16,17,18]. Identification of these components in complex mixtures requires sensitive and selective analytical techniques. Liquid chromatography coupled with spectrophotometric and mass-spectrometric detectors (LC-UV-Vis-MS) has been proven to be a useful tool for analysing works of art, especially those containing natural organic dyestuffs [19,20,21,22].

A historical carpet originating from a Turkish workshop active in the second half of the 16th century in Cairo and preserved currently in the National Museum in Kracow, Poland, was comprehensively studied in the present work. Twelve fibre samples were analysed in order to identify the natural dyes and mordants used for their manufacture. A mild extraction method with hydrofluoric acid has been optimised for dyestuffs isolation from the wool samples. The dyes were identified by high-performance liquid chromatography-mass spectrometry with atmospheric pressure electrospray ionisation in the negative mode (HPLC-ESI(-)-MS) and confirmed by quadrupole time-of-flight (QTOF) mass spectrometry. Twenty-six dyestuffs were detected and recognised or tentatively characterised, of which four compounds had not been described before. The HPLC-MS technique was also applied to analyse reference dyestuff in the extracts of weld (*Reseda luteola* L.), lac dye (*Kerria lacca* Kerr), and indigo (*Isatis tinctoria* L.) in order to provide indications of the structures of colouring substances that were detected in the historical samples but are not commercially available in pure form. Mordant ions identification was performed by SEM-EDS. Fourier transform infrared spectroscopy was utilised for substrate characterisation. The results reported in the present paper enabled full dye fingerprints in the fibre samples.

## 2. Materials and Methods

### 2.1. Chemicals and Materials

Acetonitrile and methanol used as mobile phase components of HPLC grade were purchased from Merck (Darmstadt, Germany). Hydrofluoric acid (HF, 48% in water) was purchased from Sigma-Aldrich (Steinheim, Germany). Dimethyl sulfoxide (DMSO, ACS grade) was obtained from Merck KGaA (Darmstadt, Germany). Standards of dyes: luteolin, apigenin, fistein, and luteolin 7-*O*-β-d-glucoside of HPLC purity were purchased from Sigma-Aldrich. Raw dyestuff materials: weld (*R. luteola*), lac dye (*K. lacca*), and indigo (*I. tinctoria*) were obtained from Kremer Pigmente (Aichstetten, Germany) in dried form. The dyestuff materials were homogenised prior to analysis. All aqueous solutions were prepared using deionised Milli Q water.

### 2.2. Origin of Textile Fibre Samples

**Figure 1:**
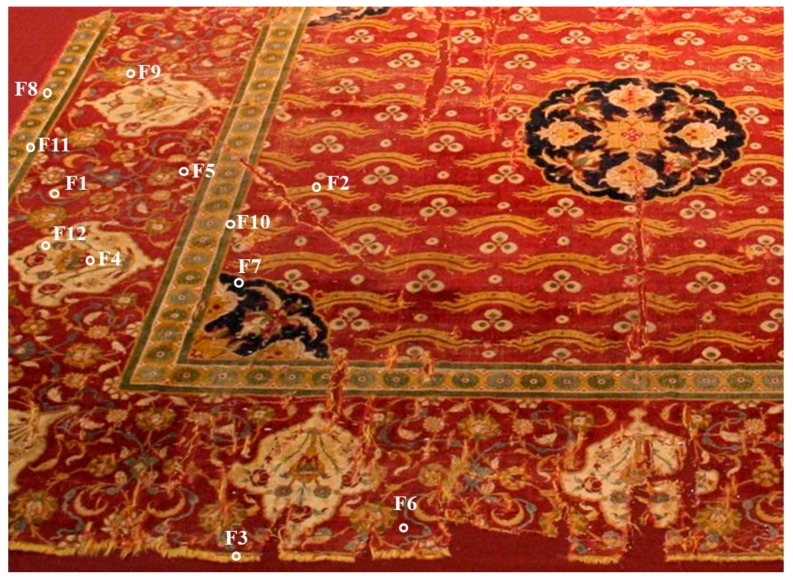
Fragment of historical carpet with fibre sampling location (National Museum in Kracow, collection MNK XIX-8950).

The samples of textile fibres were collected from a historical carpet exhibited in the National Museum in Kracow (Poland). The carpet, with Chintamani motifs, is unique because it is one of the largest preserved carpets in the world; it has a surface area of almost 40 square meters, being 1063 cm long and 372 cm wide (Figure 1). The origin of the rug was attributed to a Turkish workshop active in the second half of the 16th century in Cairo, based on the analysis of the weave structure, identification of motifs, and the technique used. According to church tradition, the carpet was donated by Stanislaw Jablonowski, the colonel of Polish King Jan Sobieski (1629–1696), after his return from the victorious Battle of Vienna (1683). In 1901, it was transferred from the Corpus Christi Church in Kracow to the collection of the National Museum. The carpet survived in eight parts, which cannot be explained merely by the structure of the warp, weft, and Persian knots breaking as a result of damage during usage. Some regular line intersections indicate purposeful extraction of parts, perhaps for the needs of the owner or collectors of works of art. One of the detached fragments is now in the collection at the Munich Museum of Ethnology.

Twelve fibre samples were collected from different parts of the carpet and are referred to as: F1, F2, F3, and F4 (yellowish of various shades), F5 and F6 (light and dark reds), F7 (navy blue), F8, F9, and F10 (greens of different shades), F11 (brown), and F12 (beige).

### 2.3. Extraction of Dyes from Threads

Dyes were extracted from the thread samples (estimated weight 0.2 mg) in an ultrasonic bath for 1 h (4 × 15 min) at a temperature not exceeding 40 °C using 500 μL of solution containing 0.4 M hydrofluoric acid/methanol/acetonitrile/DMSO (2:1:1:1, *v*/*v*/*v*/*v*). The mixtures were centrifuged at 9000 rpm for 5 min, and the supernatants were evaporated almost to dryness under a stream of nitrogen. The residues were dissolved in 300 µL of ACN/MeOH/DMSO, (1:1:1, *v*/*v*/*v*), out of which 2 μL were injected into the HPLC column. The plant raw materials used as reference for the analysis of the historical textile samples were also extracted according to the HF procedure. Standards used for identification purposes were dissolved in an ACN/MeOH/DMSO (1:1:1, *v*/*v*/*v*) mixture.

### 2.4. Equipment

The morphology of the samples was studied with an scanning electron microscopy (SEM) operated with a secondary electron detector in high vacuum mode at an accelerating voltage of 10–20 kV (FEI Quanta FEG 250, Thermo Fisher Scientific, Waltham, MA, USA). Identification of elements was performed using energy dispersive spectroscopy (EDS) on EDAX Genesis APEX 2i (Ametek, Berwyn, PA, USA) with an ApolloX SDD spectrometer at an accelerating voltage 20 kV.

Infrared transmission spectra were recorded with a Nicolet iS50 FT-IR spectrometer (Thermo Fisher Scientific, Waltham, MA, USA) equipped with the Specac Quest single-reflection diamond attenuated total reflectance (ATR) accessory. Spectral analysis was controlled by the OMNIC software package (Thermo Fisher Scientific, Waltham, MA, USA).

Analyses were performed using the liquid chromatograph series 1290 (Agilent Technology, Waldbronn, Germany) consisting of the binary pump G4220A, the autosampler G4226A, the thermostated column compartment G1316C, the diode-array detector G1315C, and the triple quadrupole mass spectrometer G6470 with an Agilent Jet Stream electrospray ionisation source. The chromatographic system was controlled with Agilent MassHunter software (B.07.05, Agilent, Santa Clara, CA, USA). The components of the extracts were separated on a C-18 reversed-phase column. The analytes were monitored with a diode array detector and a mass spectrometer connected in-line and characterised with their retention times, UV-vis, and mass spectra. In order to ensure universal elution conditions for chemically different compounds, a wide gradient of methanol/acetonitrile and water was used. The structures of the identified dyes were confirmed by HPLC-ESI(-)-QTOF analysis using an Agilent 1290 LC system coupled to the Agilent QTOF mass spectrometer G6540 (Santa Clara, CA, USA) operated in negative ionisation scan mode under the same chromatographic conditions. The parameters of the optimised spectrochromatographic analysis are presented in Table S1.

## 3. Results and Discussion

### 3.1. Microscopic and Spectroscopic Studies

#### 3.1.1. Surface Morphology

**Figure 2:**
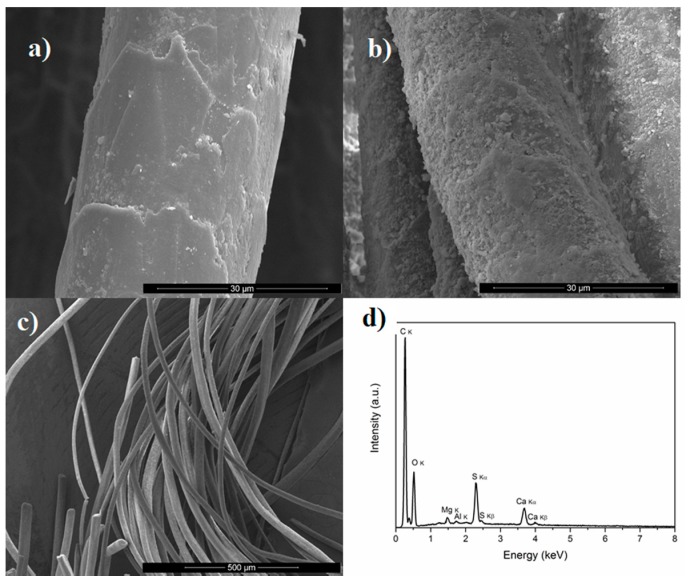
Scanning Electron Microscopy (SEM) micrographs of: (**a**) fibre F1 (magnitude 2500×); (**b**) fibre F11 (magnitude 2500×); (**c**) fibre F1 (magnitude 100×); (**d**) Scanning Electron Microscopy-Energy Dispersive Spectroscopy (SEM-EDS) spectrum of fibre F1.

Surface profiles, nature, homogeneity, and microstructure of the samples of the historical fibres were analysed using a scanning electron microscope. SEM micrographs of fibre surfaces display a cylindrical shape and nodular thickening across their length, all of which are typical for the scale structure of wool (Figure 2a–c). In some cases, roughened surfaces were observed with damage of this scale structure due to natural aging (Figure S1). Some of the samples were more degraded, since they have a greater number of fractured fibrils. The diameters of the wool fibres ranged from 20 to 40 μm.

#### 3.1.2. Fourier Transformation Infrared Spectroscopy Analysis

Infrared is a highly suitable technique for fibres characterisation and allows us to distinguish their origin [23]. The IR spectra of all fibres (Figure S2) showed a broad stretching band of amino -NH and phenolic -OH groups at 3275 cm^−1^. Absorption at 2925 and 2880 cm^−1^ was due to the C-H asymmetric stretching of aliphatic carbon compounds. The IR peak at 1225 cm^−1^ was due to C-N stretching. The weak absorption band near 1040 cm^−1^ was attributed to the presence of ether linkages. Amide I (1700–1600 cm^−1^) and amide II (1550–1500 cm^−1^) bands, which are typical for proteins, were also observed in the IR spectra of all the investigated samples. An amide I band together with weaker contributions around 1674–1695 were generally attributed to β-sheet structures. The IR band components in the 1662–1686 cm^−1^ region reflect the contribution of β-turn. Thus, the samples were unequivocally animal fibres [24]. Moreover, observation of the 640–650 cm^−1^ and 590–525 cm^−1^ bands, designated to ν (C-S) stretching vibrations, indicates that the thread samples are woollen fibres [25].

#### 3.1.3. Scanning Electron Microscopy with Energy-Dispersive X-ray Detector Analysis

SEM-EDS was employed to identify the mordant metals used during the dyeing of the historical threads. A typical EDS spectrum of the textile specimens is given in Figure 2d. Two elements, i.e., carbon and oxygen, arising from wool proteins were detected in large quantities (Table 1). Wool is mostly composed of keratin (up to 75%), which includes carbon (50%), oxygen (22%), nitrogen (17%), hydrogen (7%), and sulphur (4%) [26]. The presence of Al, Fe, S, Ca, Mg, Si and trace amounts of copper were also found. Aluminium and iron probably originate from a mordant essential to obtain fast colours. While aluminium salts do not change the colours of dyed textiles, iron and copper salts cause a darkening of yellow and red mordant dyes and tannins affect the final colour of the textile fragments during the dyeing process [26]. Calcium, Si, and Mg might originate from the contaminations during storage and utilisation of the carpet; thus, it is not possible to conclude if they are the components of the mordants. The presence of sulphur is not surprising, since this element is found in animal fibres. Chromium and potassium were not detected in any of the investigated samples, although their salts could have been used as mordants in the past [27,28]. The investigated samples did not differ significantly with respect to elemental composition, although relative amounts of particular elements were variant. The brown fibre F11 was probably covered by the greatest amount of iron and calcium salts, as can be seen in Figure 2d and Table 1. The EDS spectrum of this sample exhibits also the highest content of sulphur and silica.

### 3.2. High-Performance Liquid Chromatography-Mass Spectrometry Analysis

Identification of colouring compounds was performed by comparing their retention times and UV and mass spectra in the negative ionisation mode (ESI(−)-MS and MS/MS) to those obtained for the compounds found in weld (*Reseda luteola* L.), lac dye (*Kerria lacca* Kerr), and indigo (*Isatis tinctoria*) extracts and standards of flavonoids under the same chromatographic conditions (see experimental section). Identification of standardless and unknown colouring substances was supported by high-resolution QTOF spectra.

#### 3.2.1. Yellow Fibres

Four historical fibres possess a yellow hue. Figure 3 shows the chromatograms obtained for the extracts of the fibres: (a) F1, (b) F2, (c) F3, and (d) F4. The retention times, maximum absorbance wavelengths (λ_max_), computed elemental compositions, molecular ions, main fragment ions, and proposed formulae for the compounds are summarised in Table 2. The chemical structures and MS spectra of all of the identified compounds are presented in the supplementary information supplementary information (Figures S3–S32).

**Figure 3:**
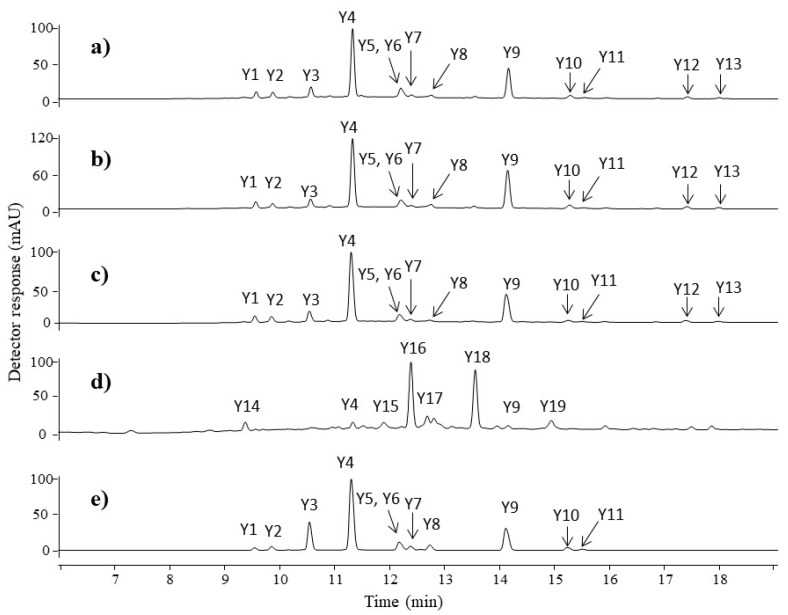
Chromatograms at 350 nm of yellow extracts taken from: (**a**) fibre F1; (**b**) fibre F2; (**c**) fibre F3; (**d**) fibre F4; and (**e**) weld raw source. For chromatographic conditions, see Table S1.

The chromatographic profiles obtained for the F1, F2, and F3 samples are quite similar and comparable to the chromatogram of the weld extract (Figure 3e). Eight flavone-glucosides and five aglycons were identified in the yellow fibres. The first compound (**Y1**), eluting at 9.5 min, was recognised as apigenin-*C*-diglucoside. It showed a pseudo-molecular ion [M−H]^−^ at *m*/*z* 593 and main fragmentation patterns at *m*/*z* 503 [M−H–90]^−^, *m*/*z* 473 [M−H–120]^−^, and *m*/*z* 575 [M−H–18]^−^. The losses of 120 and 90 units correspond to cross-ring cleavage in the sugar moiety characteristic for *C*-glycosides, whereas the ion at *m*/*z* 575 is formed by a neutral loss of H_2_O molecule. The hypothesis was confirmed by an ESI(−)-QTOF product ion mass spectrum, in which the peak [M−H]^−^ was observed at *m*/*z* 593.1513 (corresponding to the elemental composition of C_27_H_30_O_15_, mass diff. −0.17 ppm). Compound **Y2** with a pseudo-molecular ion [M−H]^−^ at *m*/*z* 609 fragmented to an [M−H–162]^−^ ion at *m*/*z* 447 and an [M−H–162–162]^−^ ion at *m*/*z* 285 (aglycone). The fragment ions correspond to the loss of one glucose and two glucose moieties from luteolin glucoside. This compound was defined as luteolin-*O*-diglucoside.

**Figure 4:**
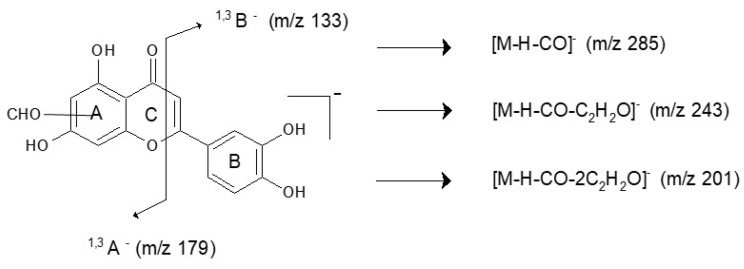
Fragmentation pathway of flavone molecules **Y12** and **Y13**.

Compound **Y3** with a pseudo-molecular ion at *m*/*z* 609 was fragmented by the loss of two glucose moieties, leading to ions at *m*/*z* 447 and *m*/*z* 285 (aglycone). The high intensity of the [M−H–162]^−^ ion suggests that sugar residues are bounded at different aglycone positions [29,30]. The compound was identified as luteolin-3,7′-*O*-diglucoside based on UV spectra and data available in the literature [31,32]. Compound **Y4**, as well as compound **Y8**, were attributed to isomers of luteolin-*O*-glucoside. Both compounds have a pseudo-molecular ion at *m*/*z* 447, which loses glucose residue (162 Da), leading to an aglycone ion at *m*/*z* 285. An ion at *m*/*z* 284 (aglycone-H)^•−^ observed in the product ion *m*/*z* 285 mass spectrum of compound **Y4** is known in the literature as a marker for distinguishing luteolin-7-*O*-glucoside (**Y4**) and luteolin-4′-*O*-glucoside (**Y8**) [33]. Identification of luteolin-7-*O*-glucoside was straightforward, as this compound is available in pure form. The chromatographic peak at retention time 12.2 min contains two co-eluting compounds **Y5** and **Y6**. The first of them was considered to be luteolin-*O*-glucoside with a pseudo-molecular ion [M−H]^−^ at *m*/*z* 447 and a fragment ion at *m*/*z* 285 (aglycone). The second component (**Y6**) was attributed to apigenin-7-*O*-glucoside with the precursor ion [M−H]^−^ at *m*/*z* 431 and fragment ions at *m*/*z* 311, 269, and 268. A signal at *m*/*z* 311 (^0,2^X^−^ ion resulting from the loss of 120 Da) in the MS/MS spectra of the compound **Y6** was attributed to the 0,2-cleavage of the glucose moiety, which is common for flavone-7-*O*-glucoside [33]. The compound **Y7** eluting at 12.4 min with a pseudo-molecular ion at *m*/*z* 461 and a fragment ion at *m*/*z* 299, formed after the loss of glucose moiety, was attributed to the chrysoeriol glucoside. The compounds **Y9**, **Y10**, and **Y11** were identified as luteolin, apigenin, and chrysoeriol, respectively. Mass spectra of luteolin showed characteristic ions at *m*/*z*: 257 [M−H–CO]^−^, 217 [M−H–C_3_O_2_]^−^, 199 [M−H–C_2_H_2_O–CO_2_]^−^, and 175 [M−H–C_3_O_2_–C_2_H_2_O]^−^ as well as ions corresponding to retro-Diels-Alder (RDA) fragmentation of the flavone molecule, which gave rise to two species: a ^1,3^B^−^ ion at *m*/*z* 133 and a ^1,3^A^−^ ion at *m*/*z* 151. A comparable explanation of mass fragmentation has been published by, e.g., Fabre et al. and Troalen et al. [34,35]. Apigenin showed similar RDA fragmentation pathways leading to a ^1,3^B^−^ ion at *m*/*z* 117 and a ^1,3^A^−^ ion at *m*/*z* 151. The identification of these compounds was straightforward, as luteolin and apigenin standards are available. Chryoseriol was fragmented by the loss of a CH_3_^•^ unit to an ion at *m*/*z* 284, which is characteristic for the methoxy derivative of flavones, and subsequently by the neutral loss of a CO molecule, yielding a product ion at *m*/*z* 256. The chromatographic profiles of fibres F1, F2, and F3 may suggest that weld was the source of the yellow dyes. Two additional peaks (**Y12** and **Y13**) were detected in the F1, F2, and F3 fibre extracts. Mass spectra of both compounds **Y12** (*t*_R_ 17.4 min) and **Y13** (*t*_R_ 18.00 min) showed a pseudo-molecular ion at *m*/*z* 313 and main fragment ions at *m*/*z*: 285 [M−H–CO]^−^, 243 [M−H–CO–C_2_H_2_O]^−^, 201 [M−H–CO–2C_2_H_2_O]^−^, 179 [M−H–134]^−^, and 133 [M−H–180]^−^, respectively. These compounds are not present in weld extract. Signals at *m*/*z* 179 and 133 correspond to ions ^1,3^A^−^ and ^1,3^B^−^ formed during RDA fragmentation of the flavone molecule (Figure 4). The unique fragment ion at *m*/*z* 133 is similar to the ^1,3^B fragment ion of luteolin, whereas the fragment ion at 179 *m*/*z* differs by 28 atomic mass units from the luteolin ^1,3^A^−^ ion. This difference suggests that in the A ring of compounds **Y12** and **Y13**, additional substituent containing carbon and oxygen elements is present, i.e., a formyl group. Different retention times of compounds **Y12** and **Y13** imply that this substituent is attached to different positions at the aglycone, although it is not possible to conclude the exact location. This hypothesis was confirmed by ESI(−)-QTOF product ions mass spectra, in which the pseudo-molecular ions of compounds **Y12** and **Y13** corresponding to the elemental composition of C_16_H_10_O_7_ were observed at *m*/*z* 313.0349 (mass diff. 1.6 ppm) and 313.0342 (mass diff. 3.8 ppm). To the best of our knowledge, these compounds have not been described in the literature before. Moreover, such specific molecules can be distinct markers for the Turkish workshop.

The UV chromatogram of the orange-yellow fibre F4 extract showed two main peaks at 12.4 min and 13.6 min. The peak eluted at 13.6 min (**Y18**) with a pseudo-molecular ion at *m*/*z* 269 gave fragment ions at *m*/*z*: 241 [M−H–CO]^−^, 225 [M−H–CO_2_]^−^, 213 [M−H–2CO]^−^, 195 [M−H–2CO–H_2_O]^−^, 135, and 133. Ions at *m*/*z* 135 and 133 were formed from the A and B rings of aurone. The UV spectrum of this substance had λ_max_ at 256, 275, and 396 nm. The obtained results and literature data allowed us to postulate that the detected compound is sulfuretin [36,37,38]. The peak eluted at 12.4 min, marked as **Y16**, also has ion [M−H]^−^ at *m*/*z* 269 and fragment ions at *m*/*z* 241, 225, 135, and 133, similar to **Y18**. This compound seems to be an isomer of sulfuretin; however, the exact structure is unclear. The peak **Y17**, appearing at the retention time 12.7 min with mass peak [M−H]^−^ at *m*/*z* 285 and product ions of aglycone RDA fragmentation at *m*/*z*: 241 [M−H–CO_2_]^−^, 229 [M−H–2CO]^−^, 149, 135, and 121, was identified as fisetin. The identification of this compound was straightforward, as the fisetin standard is available. Some other dyestuffs were detected in the yellow thread F4. Minor peaks in the chromatogram (Figure 3d), indicated as compounds **Y14**, **Y15**, and **Y19**, showed pseudo-molecular ions [M−H]^−^ at *m*/*z* 349, 243, and 314, respectively. The structures of these compounds remain unknown. The peak labelled as **Y15** showed the main ion at *m*/*z* 243 with fragment ions at *m*/*z*: 215 [M−H–CO]^−^, 199 [M−H–CO_2_]^−^, 187 [M−H–2CO]^−^, 175, and 113. The absorption spectrum showed maxima at 258, 308, and 336 nm. Based on the result obtained and data available in the literature, compound **Y15** was identified as the photodegradation product of brazilein, known as a “type C compound” [39,40]. Brazilwood (soluble redwood) is known for its fast light degradation, and “type C compound” is often used as a marker for the identification of the soluble redwoods in samples extracted from historical artworks [41,42]. Despite the fact that the sample F4 nowadays presents a yellowish tone, it is thought to have been originally dyed in an orange hue. The presence of sulfuretin, luteolin, and “type C compound” led us to the conclusion that the fibre F4 was dyed with young fustic, weld, and soluble redwood.

#### 3.2.2. Red Fibres

**Figure 5:**
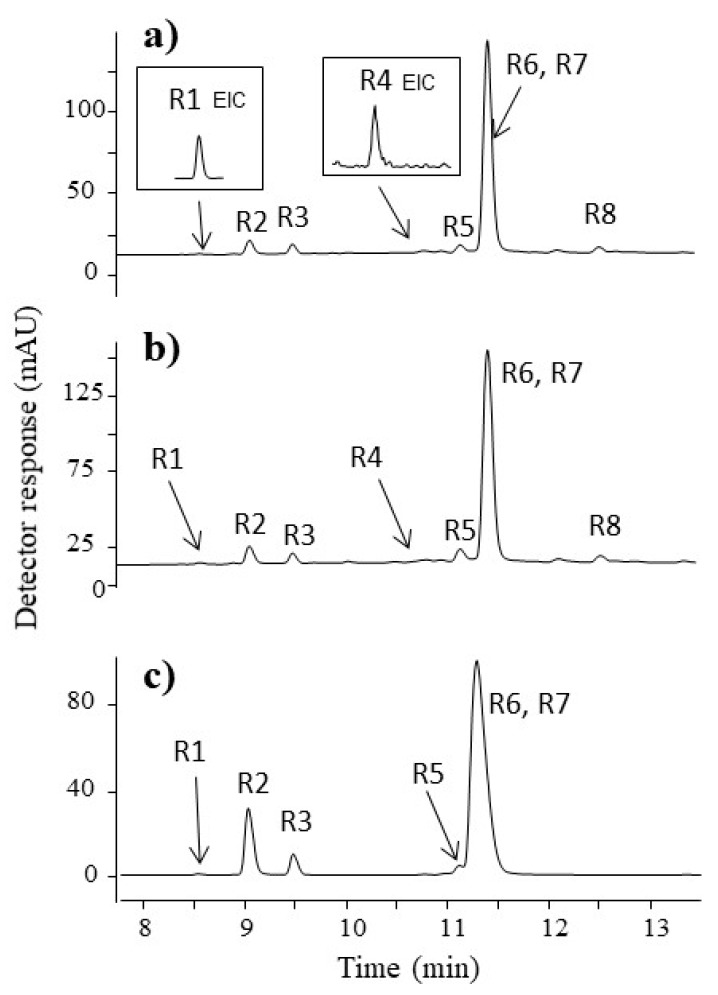
Chromatograms at 350 nm of red extracts taken from: (**a**) fibre F5; (**b**) fibre **F6**; and (**c**) lac dye raw source. For chromatographic conditions, see Table S1.

The chromatographic profiles of the red fibre extracts (F5 and F6) revealed the presence of one major peak and six minor peaks (Figure 5a–b).

The major chromatographic peak seen in UV spectrochromatograms at 11.3 min (**R6** and **R7**) had a rather complex mass spectrum. The chromatogram deconvolution algorithm of the MassHunter software revealed two co-eluting components. The reconstructed mass spectrum of the first component showed a pseudo-molecular ion at *m*/*z* 536 and the main product ions at *m*/*z* 492 and 448, while the pseudo-molecular ion at *m*/*z* 495 with the two most intensive product ions at *m*/*z* 451 and 407 can be seen in the reconstructed mass spectrum of the second component (see Table 2). The structure of the first component corresponds to laccaic acid A with fragment ions at *m*/*z*: 518 [M−H–H_2_O]^−^, 492 [M−H–CO_2_]^−^, 474 [M−H–CO_2_–H_2_O]^−^, 448 [M−H–2CO_2_]^−^, 430 [M−H–2CO_2_–H_2_O]^−^, and 420 [M−H–2CO_2_–CO]^−^ [43]. Laccaic acid B was assigned to the second component, with product ions at *m*/*z*: 447 [M−H–H_2_O]^−^, 451 [M−H–CO_2_]^−^, 433 [M−H–CO_2_–H_2_O]^−^, 407 [M−H–2CO_2_]^−^, and 389 [M−H–2CO_2_–H_2_O]^−^. Decarbonylation of one or more of the keto groups is the main fragmentation reaction of anthraquinoids [44,45]. Successive decarbonylation may yield to fragment ions [M−H–28]^−^, [M−H–56]^−^, and [M−H–84]^−^. Decarboxylation of the carboxylic acid group [M−H–44]^−^ is a fragmentation pathway typical for laccaic acids, followed by the loss of water [M−H–62]^−^. The other intensive fragment ions [M−H–88]^−^ and [M−H–106]^−^ correspond to the loss of two CO_2_ molecules and a subsequent loss of water from the fragment ion [M−H–2CO_2_–H_2_O]^−^.

Peak **R2** had a mass ion [M−H]^−^ at *m*/*z* 538 accompanied by product ions at *m*/*z* 520, 494, 476, 450, and *m*/*z* 432. The peak at *m*/*z* 538 can be attributed to the depronated molecular ion of laccaic acid C with product ions formed by the loss of H_2_O or CO_2_ molecules. The mass peak at *m*/*z* 494 (compound **R3**) accompanied by related peaks at *m*/*z*: 476 [M−H–H_2_O]^−^, 450 [M−H–CO_2_]^−^, 432 [M−H–CO_2_–H_2_O]^−^, 406 [M−H–2CO_2_]^−^, 388 [M−H–2CO_2_–H_2_O]^−^, and 378 [M−H–2CO_2_–CO]^−^ proved the presence of laccaic acid E. The chromatographic peak **R5** had the signal of the pseudo-molecular ion registered at *m*/*z* 520 and fragment ions at *m*/*z* 502, 476, 458, 432, and 414 formed by the subsequent loss of water and carbon dioxide. The observed signals indicated that the examined extract contains xantholaccaic acid A, which is a derivative of laccaic acid A after the loss of hydroxyl substituent in the anthraquinone skeleton. These results are in agreement with the data available in the literature [46]. The enlargement of the UV chromatographic trace shows a peak of low intensity at the retention time of 8.5 min (peak **R1**) eluted before the peak of laccaic acid C. The mass spectrum of this compound revealed the presence of a precursor ion [M−H]^−^ at *m*/*z* 522 and fragment ions at *m*/*z* 478 [M−H–CO_2_]^−^ and *m*/*z* 434 [M−H–2CO_2_]^−^. An accurate mass measurement of **R1**’s pseudo-molecular ion (*m*/*z* 522.0656, mass diff. 4.02 ppm) and main fragment ions (*m*/*z* 478.0762, mass diff. 3.56 ppm and *m*/*z* 434.0879, mass diff. 0.46 ppm) allowed us to identify the examined compound as the derivative of laccaic acid C formed after loss of the hydroxyl group. To our best knowledge, this derivative, named by us xantholaccaic acid C, is so far unknown in the literature and could be detected and identified as a result of the newly developed analytical protocol. Peak **R4**, appearing at the retention time 11.0 min, showed a pseudo-molecular ion [M−H]^−^ at *m*/*z* 552 that generated fragment ions at *m*/*z* 534 [M−H–H_2_O]^−^, *m*/*z* 508 [M−H–CO_2_]^−^, *m*/*z* 490 [M−H–CO_2_–H_2_O]^−^, *m*/*z* 464 [M−H–2CO_2_]^−^, and *m*/*z* 446 [M−H–2CO_2_–H_2_O]^−^. This compound was tentatively identified as a derivative of laccaic acid A enriched by one additional hydroxyl group. This hypothesis was confirmed by the ESI(−)-QTOF product ion mass spectrum with the pseudo-molecular peak of [M−H]^−^ at *m*/*z* 552.0769 corresponding to the elemental composition of C_26_H_19_NO_13_ (mass diff. 2.54 ppm) and a fragment ion at *m*/*z* 534.0675 (C_26_H_17_NO_12_, mass diff. 0.56 ppm), although it is not possible to conclude the exact position of this hydroxyl substituent. This compound was detected only in the naturally aged fibre samples but not in the lac dye extract. It was probably formed from its precursor, laccaic acid A (compound **R6**), during the dyeing process or as a consequence of aging. To the best of our knowledge, this colouring compound has not so far been reported in the literature and can be treated as a new dating or locating marker of historical carpets. A comparison of the HPLC profiles of the red fibre and lac dye extracts (Figure 5c) allows us to conclude that *K. lacca* insect has been used in the dyeing process of these threads.

#### 3.2.3. Blue and Green Fibres

**Figure 6:**
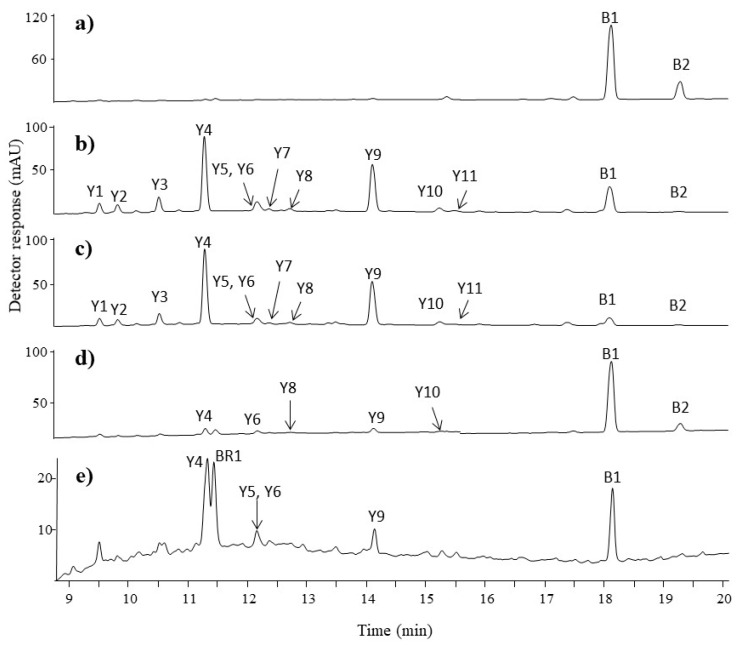
Chromatograms at 350 nm of blue, green, and brown extracts taken from: (**a**) fibre F7; (**b**) fibre F8; (**c**) fibre F9; (**d**) fibre F10; and (**e**) fibre F11. For chromatographic conditions, see Table S1.

HPLC-ESI(−)-MS of the blue fibre extract (F7) indicated the presence of two compounds **B1** and **B2** (Figure 6a). Compound **B1** with a pseudo-molecular ion [M−H]^−^ at *m*/*z* 261, and two less intensive fragment ions at *m*/*z* 233 [M−H–CO]^−^ and 217 [M−CONH_2_]^−^, was identified as indigotin. Compound **B2**, defined as indirubin, showed the same pseudo-molecular ion ([M−H]^−^ at *m*/*z* 261) and a similar fragmentation pathway. Fortunately, indigotin and indirubin can be easily differentiated due to their different retention times (18.1 and 19.2 min) and absorption spectra in the visible region. Indigotin has maximum absorbance at 620 nm whereas indirubin has maximum absorbance at 550 nm. The detection of these colouring substances suggests that indigo was used for dyeing, but it is not possible to determine what kind of plant (*Isatis tinctoria* L. or *Indigoferia tinctoria* L.) was used during the dyeing process.

Extracts of three fibres (F8, F9, and F10) exhibited a green hue. UV chromatograms of the F8 and F9 samples were very similar (Figure 6b–c). Eleven flavonoids were detected in these extracts. The presence of luteolin (**Y9**), apigenin (**Y10**), chryoseriol (**Y11**), and its glycosides (see Table 2, compounds **Y1**–**Y8**) confirms the use of weld in the colouring process. Both vat dye constituents, indigotin and indirubin, were also detected. Due to the fact that natural green dyes are rare, the green hues were usually obtained by sequential dyeing with blue and yellow dyes [47]. This was confirmed by the presence of blue indigoids and yellow flavonoids (luteolin-7-*O*-glucoside, apigenin-7-*O*-glucoside, luteolin-4′-*O*-glucoside, luteolin, and apigenin) in all of the green fibre extracts. Many historical recipes refer to the use of indigo and weld to dye wool fibres in green hues and that combination has already been reported in the literature [23,32]. As expected, the higher amount of indigotin is related to the darker green hue of sample F10 in comparison to fibres F8 and F9.

#### 3.2.4. Beige and Brown Fibres

The fibre designated as F11 has a bronze hue. Brown shades were obtained by the use of three types of dyes. Traces of “luteolin-type” flavonoids were detected in this wool extract: luteolin-7-*O*-glucoside (Y4), luteolin-*O*-glucoside (Y5), apigenin-7-*O*-glucoside (Y6), and luteolin (Y9) (Figure 6e). The dyestuff composition suggests the use of weld, but chrysoeriol was not detected; therefore, the use of another luteolin-containing plant may not be excluded [48]. The presence of ellagic acid (**BR1**), indigotin (**B1**), and indirubin (**B2**) indicates the use of a tannin-containing plant material and indigo, either for textile dyeing or for weighting the wool [27]. Tannins in combination with iron mordants were frequently used in the past to achieve a brown or black hue of threads [27,28,42]. This procedure could explain the highest amount of iron detected in the fibre F11 by SEM-EDS (Table 1). Thread F12 probably was not pigmented, because not even a trace amount of dyes was found in it.

## 4. Conclusions

A multi-analytical approach combining HPLC-UV, HPLC-MS, FT-IR, and SEM-EDS was fundamental to the successful identification and characterisation of substrates, mordants, and colouring substances present in the historical carpet. SEM micrographs of the fibres display a cylindrical shape with nodular thickening across their length, which are characteristic for the scale structure of animal wool. Two elements, i.e., carbon and oxygen, were detected in large quantities with SEM-EDS. They are part of wool proteins. All of the fibre samples contained aluminium, iron, and calcium, originating probably from mordants. EDS analyses also revealed the existence of small traces of copper, silica, and magnesium. However, they were assumed to be a contamination from the vats employed in the wool treatment, as they were barely detected. Mild conditions of dyestuffs extraction allowed us to acquire the full fingerprint of the yellow, red, blue, brown, and green dye sources, allowing us to comprehend how the final colours were obtained. High resolution mass spectrometry facilitated the identification of a number of colour organic compounds present in the wool fibre extracts, while characteristic fragmentation pathways provided additional information on the structures of the analytes. Four thus-far unknown compounds were found and identified. These molecules were discovered, identified, and confirmed for the first time as a consequence of the effective extraction method. Two luteolin derivatives (compounds **Y12** and **Y13**) were detected in yellow fibres. A derivative of laccaic acid C—“xantholaccaic acid C” (compound **R1**) and a derivative of laccaic acid A (compound **R4**), enriched by one hydroxyl group within the anthraquinone skeleton, were detected in the red fibre extracts. Weld was identified as the dye source in the yellow thread samples. The yellow-orange hue of the F4 sample originates from coloration with young fustic and weld. Although the analysis of the F4 fibre extract did not reveal the presence of brazilein and hematein, identification of the “type C compound” marker in the extract confirms the use of soluble redwood as the dyeing source as well. Red fibres have been coloured with lac dye. The green fibre shades were obtained with indigo and weld in two baths. The brown wool fibre was dyed with weld, indigo, and tannin-containing plant material.

With this study, it was possible to contribute to a better understanding of what materials and techniques were used for the carpet’s production. Knowledge of historical artwork components shall enable appropriate preservation of textile treasures with the original materials and methods.

## Figures and Tables

**Table 1:** Composition of elements (in atomic % ^a^) based on EDS analysis.

Fibre No.	F1	F2	F3	F4	F5	F6	F7	F8	F9	F10	F11	F12
Element												
C	70	64	76	67	72	66	71	64	62	65	58	69
O	27	33	22	31	27	32	27	34	36	33	33	26
Al	0.5	0.6	0.2	0.1	0.1	0.3	0.1	0.3	0.3	0.3	0.6	0.5
Si	0.2	0.3	-	0.1	-	0.1	0.1	0.4	0.4	0.1	0.9	0.6
S	0.8	0.5	1.0	0.7	0.7	0.7	0.6	0.5	0.6	0.5	3.5	2.7
Ca	0.5	0.5	0.4	0.4	1.0	0.4	0.5	0.3	0.5	0.4	2.1	0.6
Fe	<0.1	<0.1	-	0.15	0.2	0.1	<0.1	<0.1	<0.1	<0.1	1.6	0.1
Mg	0.2	0.2	0.2	0.1	<0.1	<0.1	0.1	<0.1	0.1	<0.1	0.2	0.3
Na	-	0.5	-	-	-	-	-	-	-	-	-	0.5
Traces ^b^	P, Cu	P	Cu	Cu	Cu	Cu	-	Cu	P, Cu	P	-	Cu

^a^ Uncertainty of oxygen and carbon is ±3%, for other elements ±0.1%; ^b^ elements detected below 0.1% have been labelled as “traces”.

**Table 2:** Spectrochromatographic data of the components extracted from all historical fibres.

Peak No.	*t*_R_ (min)	[M−H]^−^, *m*/*z*		MS^2^ Product Ions (*m*/*z*)	Elemental Composition	Diff (ppm)	Proposed Identification	χ_max_ (nm)
		Nominal	Highly Resolved					
**Y1**	9.6	593	593.1513	503, 575, 473, 383	C_27_H_30_O_15_	−0.17	apigenin-*C*-diglucoside	272, 335
**Y2**	9.9	609	609.1442	447, 285	C_27_H_30_O_16_	3.12	luteolin-*O*-diglucoside	268, 336
**Y3**	10.6	609	609.1462	447, 285	C_27_H_30_O_16_	−0.16	luteolin-3,7′-*O*-diglucoside	268, 341
**Y4**	11.3	447	447.0923	285, 284	C_21_H_20_O_11_	2.24	luteolin-7-*O*-glucoside	268, 349
**Y5**	12.2	447	447.0932	285	C_21_H_20_O_11_	0.24	luteolin-*O*-glucoside	268, 337
**Y6**	12.2	431	431,0989	311, 269, 268	C_21_H_20_O_10_	−1.16	apigenin-7-*O*-glucoside	266, 348
**Y7**	12.4	461	461,1072	341, 299, 284, 283 *	C_22_H_22_O_11_	3.68	chryoseriol-*O*-glucoside	266, 348
**Y8**	12.8	447	447.0936	285	C_21_H_20_O_11_	−0.67	luteolin-4′-*O*-glucoside	268, 342
**Y9**	14.2	285	285.0407	257, 217, 199, 175, 151, 133	C_15_H_10_O_6_	−1.05	luteolin	255, 349
**Y10**	15.3	269	269.0454	225, 151, 117	C_15_H_10_O_5_	0.37	apigenin	267, 337
**Y11**	15.5	299	299.0563	284, 256	C_16_H_12_O_6_	−0.67	chryoseriol	266, 347
**Y12**	17.4	313	313.0349	285, 243, 201, 179, 133	C_16_H_10_O_7_	1.60	luteolin derivative	248, 346
**Y13**	18.0	313	313.0342	285, 243, 201, 179, 133	C_16_H_10_O_7_	3.83	luteolin derivative	242, 346
**Y14**	9.4	349	349.0028	371, 338, 269, 225, 213, 177, 165, 149, 135, 121	-	-	unknown	261, 392
**Y15**	12.0	243	243.0294	215, 199, 187, 175, 145 *	-	-	type C	308, 336
**Y16**	12.4	269	269.0448	241, 225,185, 135, 133	C_15_H_10_O_5_	2.60	sulfuretin isomer	316, 343
**Y17**	12.7	285	285.0407	241, 229, 149, 135, 121	C_15_H_10_O_6_	−1.05	fistein	320, 360
**Y18**	13.5	269	269.0459	241, 225, 213, 195, 135	C_15_H_10_O_5_	−1.47	sulfuretin	256, 396
**Y19**	14.9	314	314.0302	267, 239, 217, 199, 163, 135	-	-	unknown	292, 342
**R1**	8.5	522	522.0656	478, 434	C_25_H_17_NO_12_	4.02	xantholaccaic acid C	293, 425
**R2**	9.0	538	538.0626	520, 494,476, 450, 432	C_25_H_17_NO_13_	0.19	laccaic acid C	288, 490
**R3**	9.5	494	494.0723	476, 450, 432, 406, 388, 378	C_24_H_17_NO_11_	1.21	laccaic acid E	288, 490
**R4**	11.0	552	552.0769	534, 508, 490, 464, 446	C_26_H_19_NO_13_	2.54	derivative of laccaic acid A	285, 504
**R5**	11.1	520	520.0854	502, 476, 458, 432, 414	C_26_H_19_NO_11_	5.96	xantholaccaic acid A	294, 430
**R6**	11.3	536	536.0836	518, 492, 474, 448, 430, 420	C_26_H_19_NO_12_	−0.37	laccaic acid A	288, 490
**R7**	11.3	495	495.0568	477, 451, 433,407, 389	C_24_H_16_NO_12_	0.2	laccaic acid B	288,490
**R8**	12.5	606	606.1184	562, 518	-	-	unknown	288, 492
**B1**	18.1	261	261.0665	233, 217	C_16_H_10_N_2_O_2_	1.53	indigotin	288, 620
**B2**	19.2	261	261.0669	233, 217	C_16_H_10_N_2_O_2_	0	indirubin	290, 550
**BR1**	11.5	301	300.9992	284, 257, 229	C_14_H_8_O_8_	2.3	ellagic acid	255, 355

* Results from first-order mass experiment, t_R_—retention time, MS^2^—second-order mass ions, Diff—mass difference, χ_max_—maximum absorbance.

## Supplementary Materials

Supplementary materials are available online. Figure S1: Compilation of SEM images of all tested threads at a magnification of 100×, Figure S2: Typical FT-IR spectra of the selected treads: A-fibre F5, B-fibre F1, Figures S3–S32: Mass spectra of all detected compounds, Figure S33: Historical carpet with Chintamani motifs (National Museum in Kracow, Poland collection MNK XIX-8950), Table S1: Conditions of chromatographic separation and detection of examined colorants.
