# Supplementary material for: Chromatographic and Spectroscopic Identification and Recognition of Natural Dyes, Uncommon Dyestuff Components, and Mordants: Case Study of a 16th Century Carpet with Chintamani Motifs
Source: Molecules. 2018 Feb 6;23(2):339. doi: 10.3390/molecules23020339 (PMC6017628; doi:10.3390/molecules23020339)
Supplement: Supplementary file 1 [file molecules-23-00339-s001.pdf]

**HPLC separation**			
Column	Poroshell EC-C18 (3.0×150 mm),<br>2.7 μm, Agilent Technologies		
Column temperature	40 °C		
Injection volume	2 μL		
Flow rate	0.4 mL min $^{-1}$		
Eluents	(A) 0.1 % HCOOH in water,<br>(B) 0.1 % HCOOH in ACN/MeOH (1:1; *v/v*)		
Gradient program	Time, min	% A	% B
	0	90	10
	2	90	10
	20	0	100
	30	0	100
Post time	10 min		
**UV–Vis detection**			
Wavelengths	UV: 254, 350 nm; Vis: 580 nm		
Peak width	> 0.1 min (2 s)		

**ESI MS detection**	**QQQ**	**QTOF**
Polarity	Negative	
Mode	Profile 50-1000 m/z<br>Product ion 50-650 m/z	50-1000 m/z
Peak width	0.07 min	-
Fragmentor voltage	200 V	100 V
Sheath gas temperature	250 °C	325 °C
Sheath gas flow	11 L min $^{-1}$	10 L min $^{-1}$
Drying gas flow	5 L min $^{-1}$	-
Drying gas temperature	300 °C	-
Nebulizer pressure	45 psi	35 psi
Capillary voltage	3500 V	3500 V
VCharging	500 V	-
Collision energy	20 V	-

Table S1. Conditions of chromatographic separation and detection of examined colorants

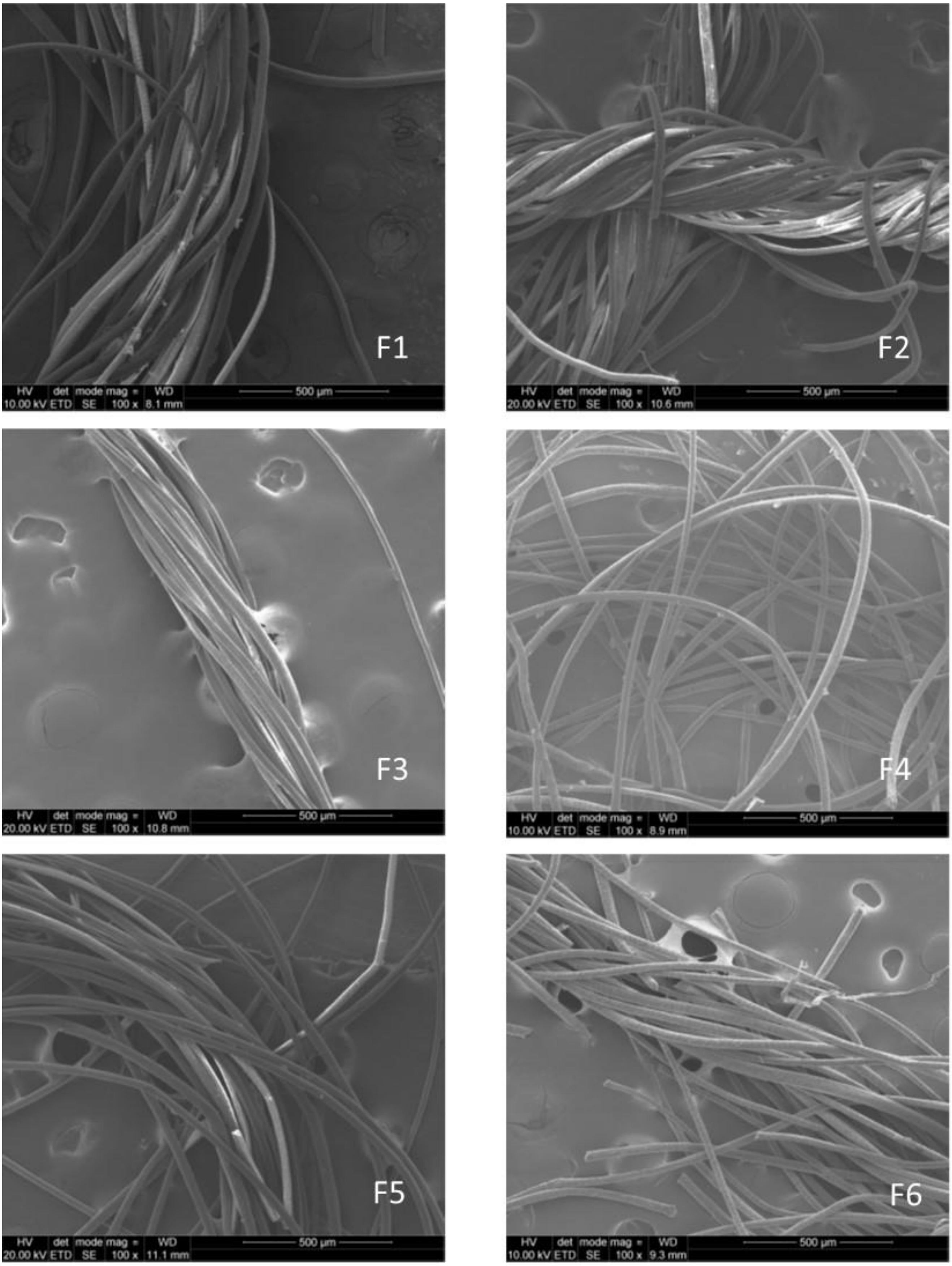

Figure S1. Compilation of SEM images of all tested threads at a magnification of 100x

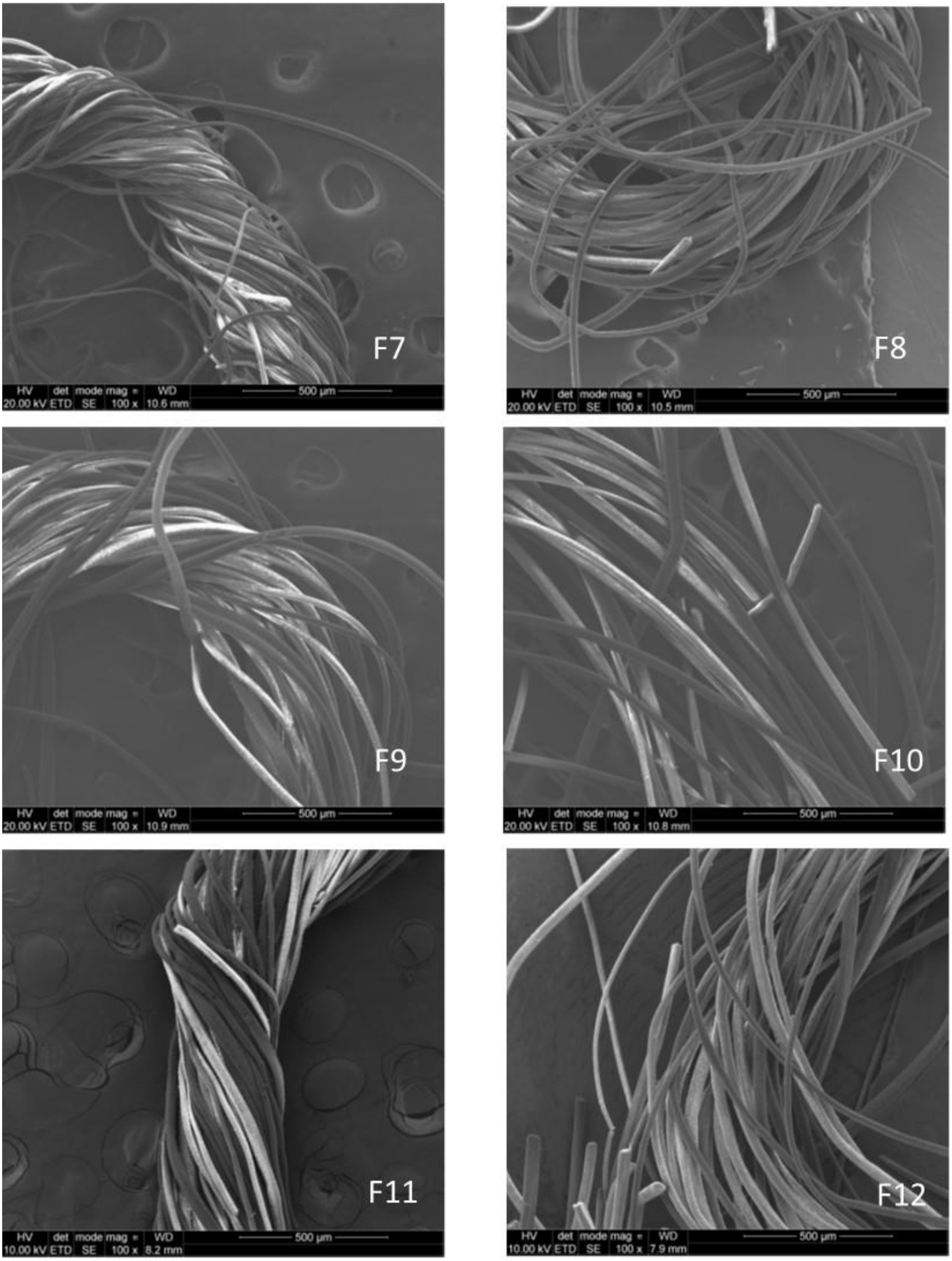

Fig.S1.(cont.) Compilation of SEM images of all tested threads at a magnification of 100x

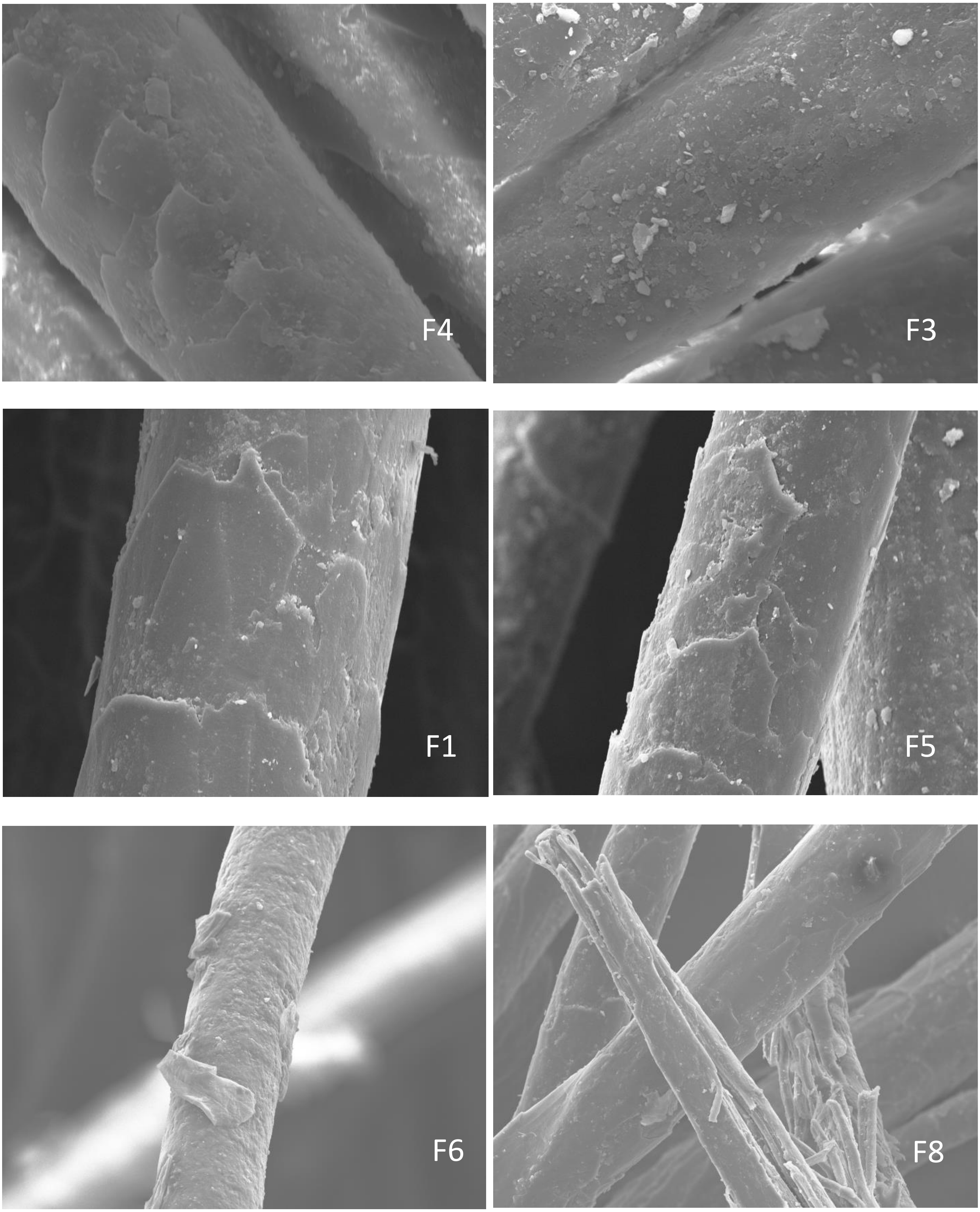

Fig.S1.(cont.) Compilation of SEM images of selected threads at a magnification of 2500x

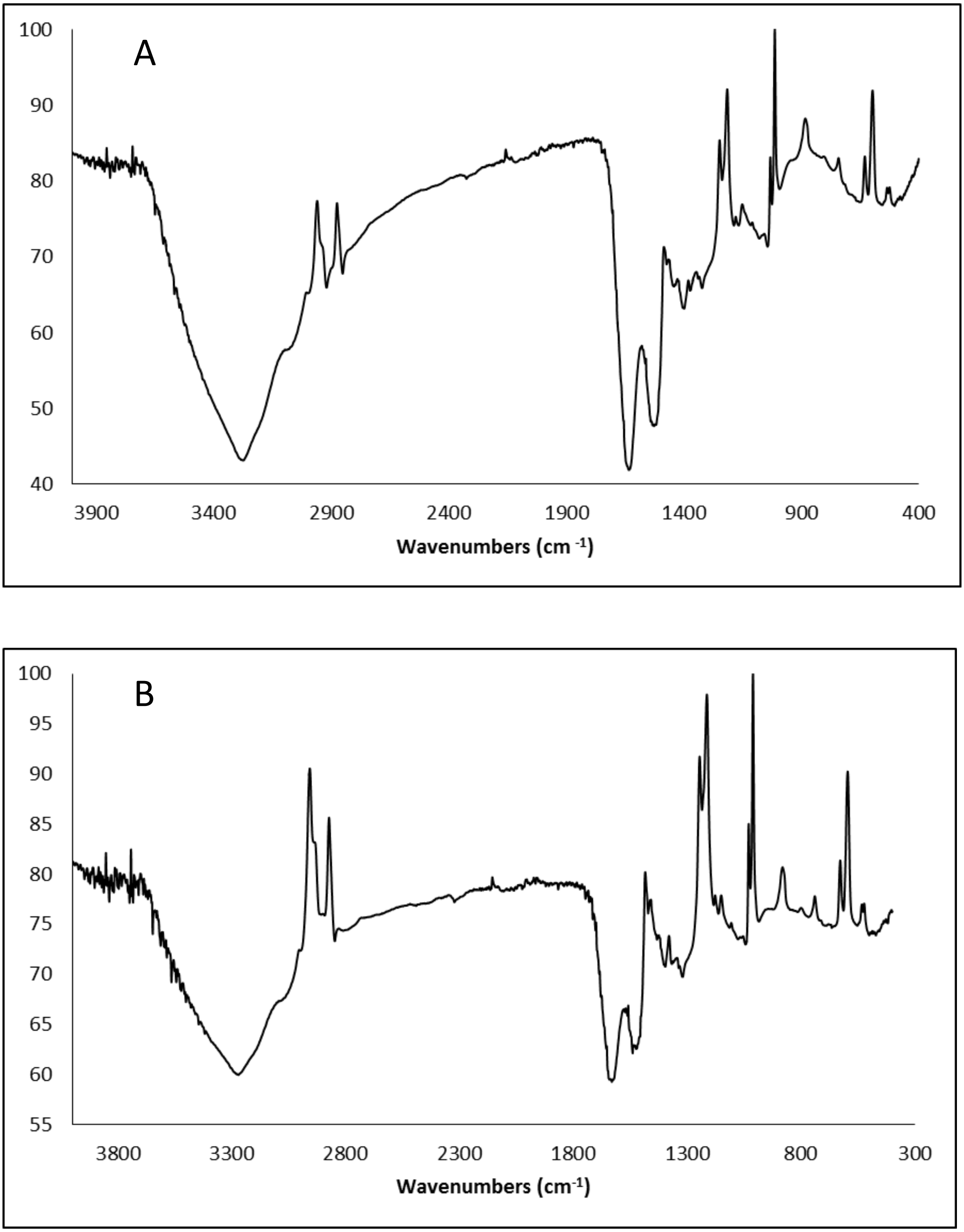

Fig.S2. Typical FT-IR spectra of the selected treads: A- fibre F5, B-fibre F1

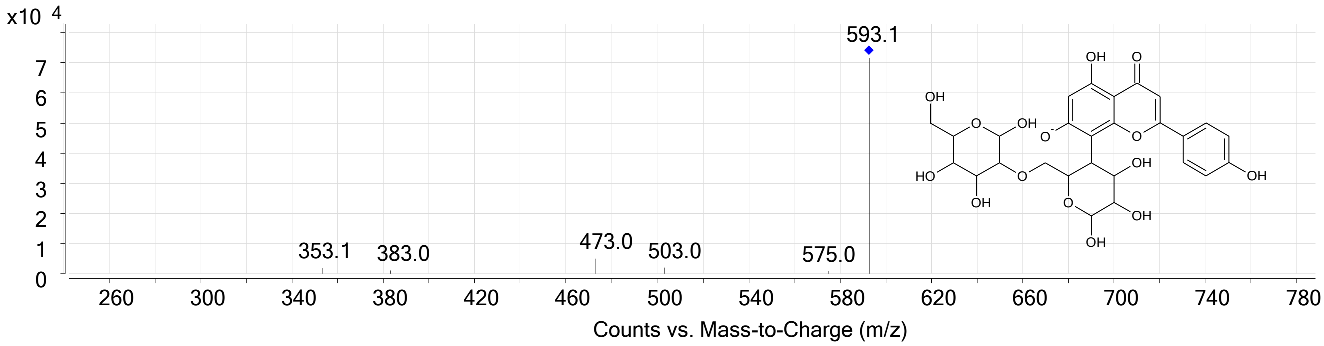

Fig. S3. Mass spectrum of apigenin-*C*-diglucoside (Y1).

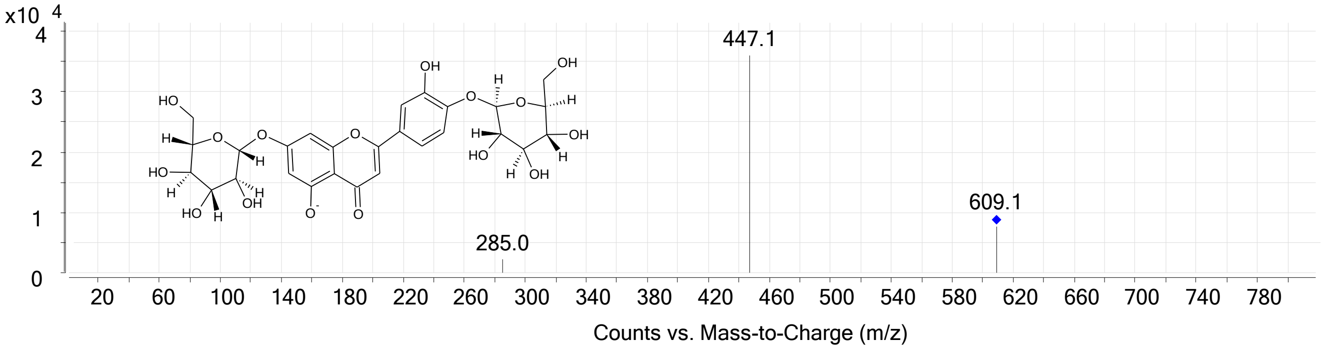

Fig. S4. Mass spectrum of luteolin-*O*-diglucoside (Y2).

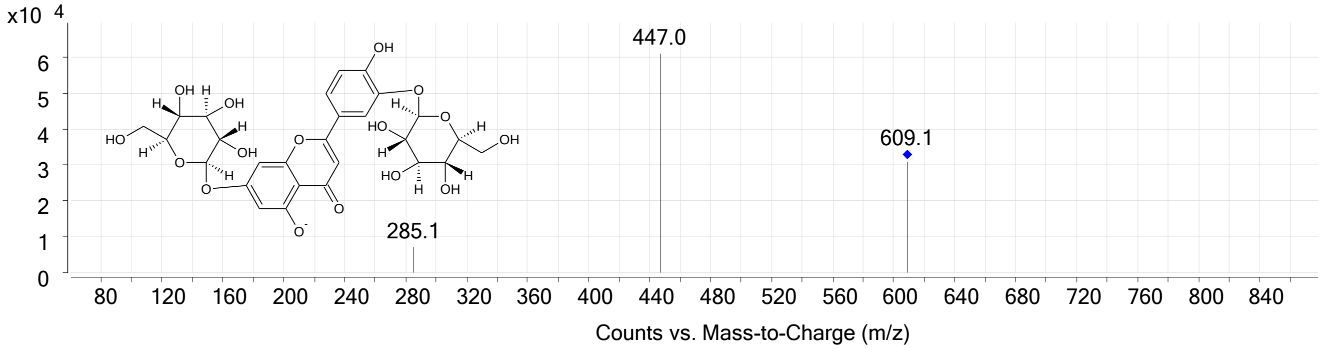

Fig. S5. Mass spectrum of luteolin-3,7'-*O*-diglucoside (Y3).

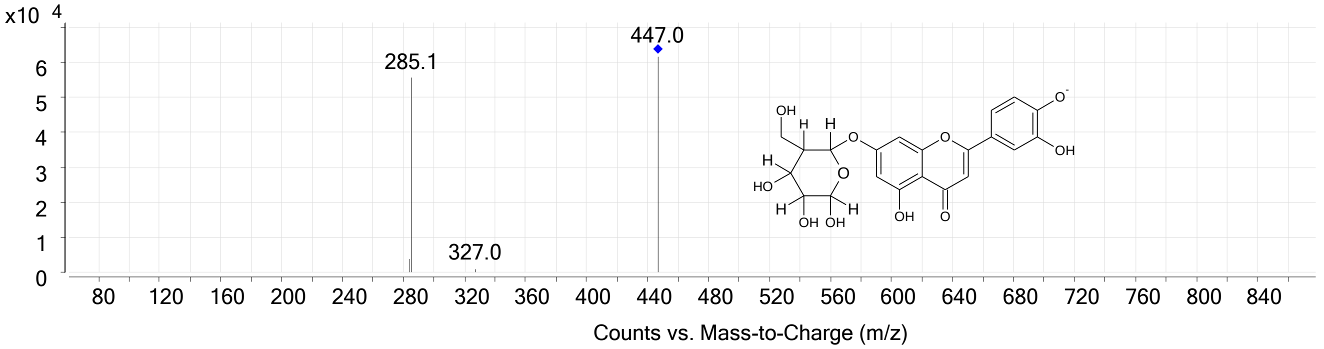

Fig. S6. Mass spectrum of luteolin-7-*O*-glucoside (Y4).

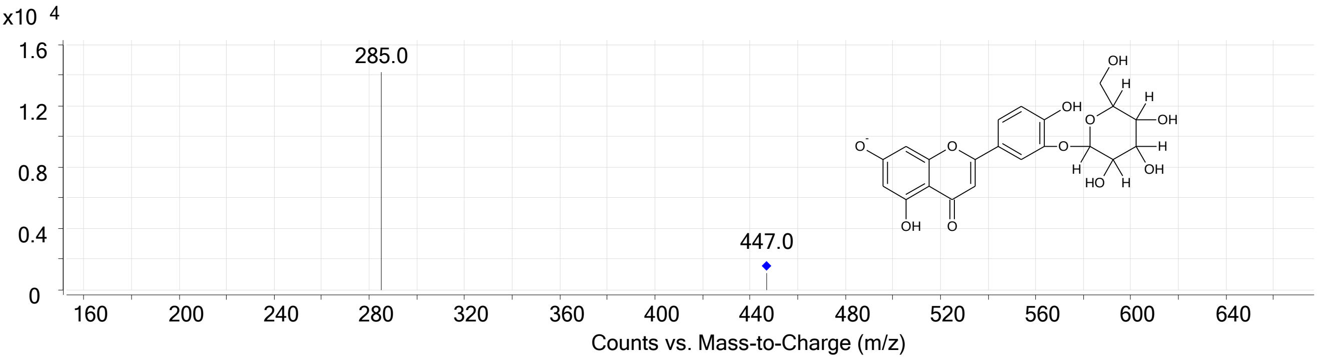

Fig. S7. Mass spectrum of luteolin-*O*-glucoside (Y5).

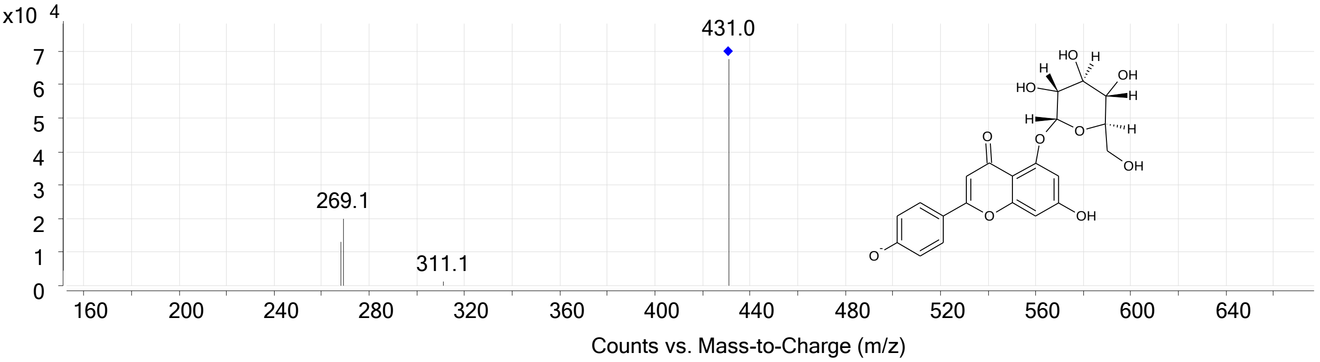

Fig. S8. Mass spectrum of apigenin-7-*O*-glucoside (Y6).

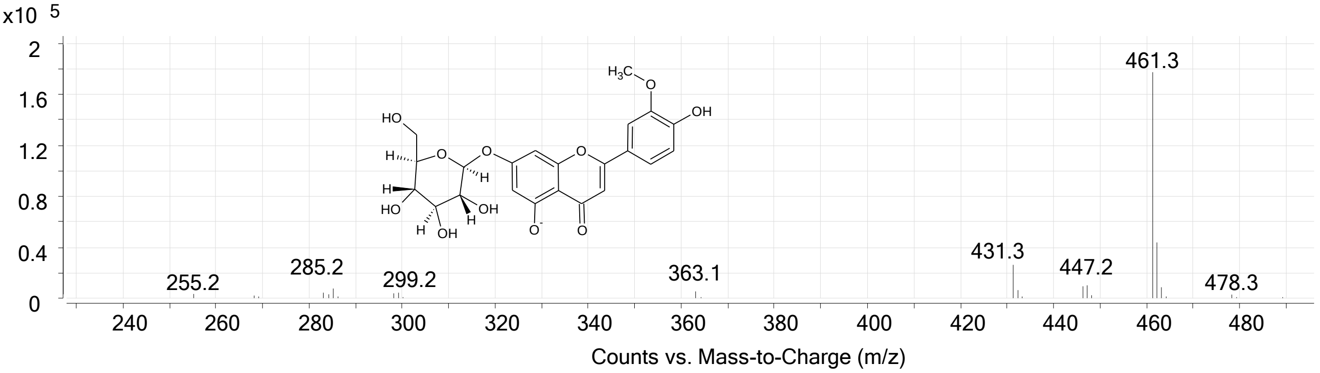

Fig. S9. Mass spectrum of chryoseriol-*O*-glucoside (Y7).

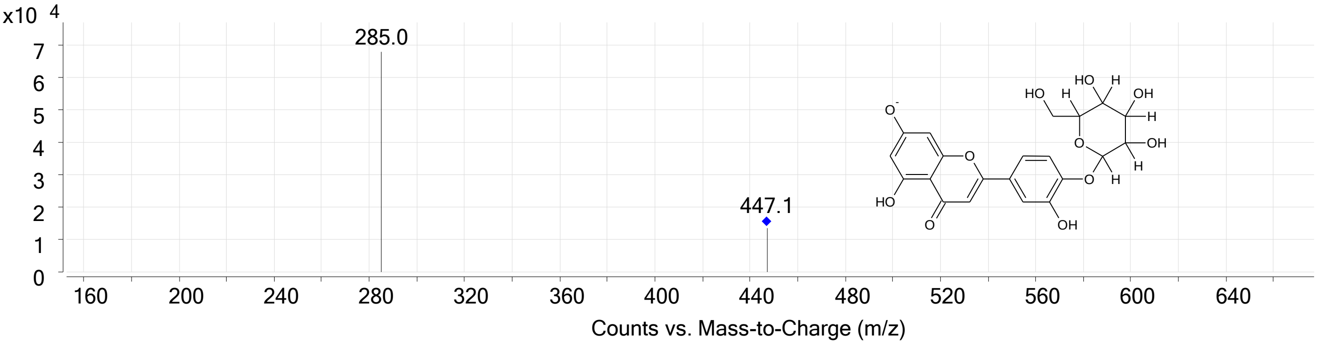

Fig. S10. Mass spectrum of luteolin-4’-*O*-glucoside (Y8).

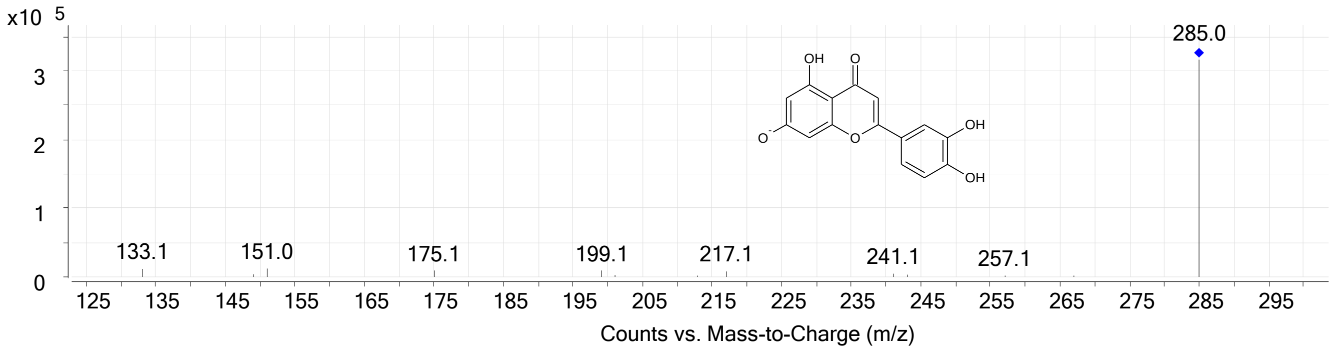

Fig. S11. Mass spectrum of luteolin (Y9).

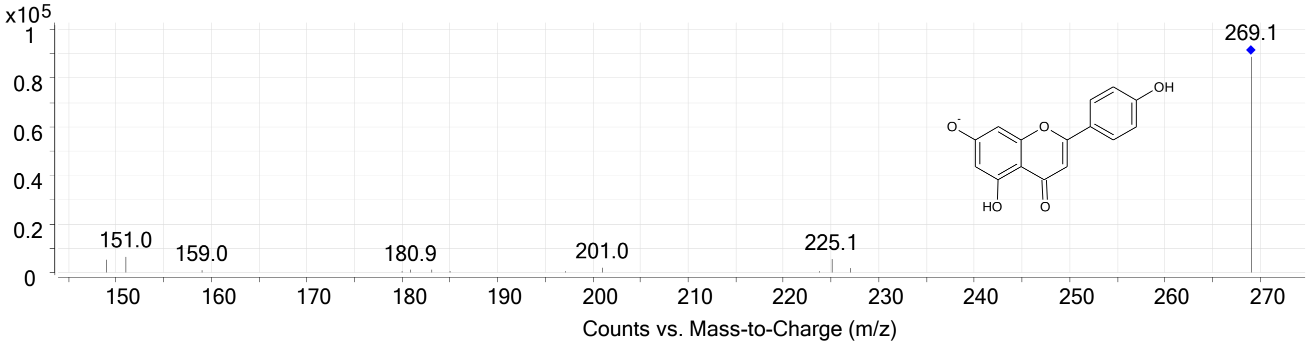

Fig. S12. Mass spectrum of apigenin (Y10).

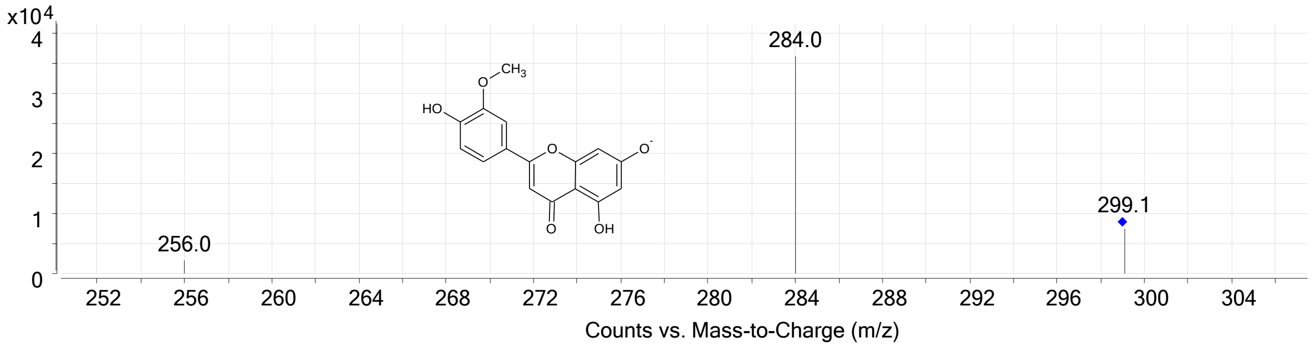

Fig. S13. Mass spectrum of chryoseriol (Y11).

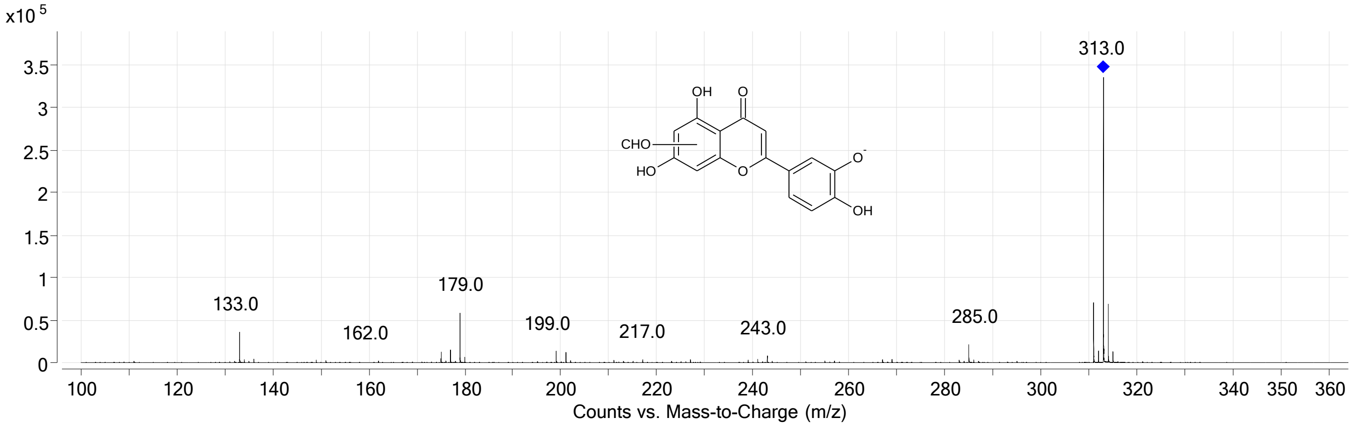

Fig. S14. Mass spectrum of luteolin derivative (Y12).

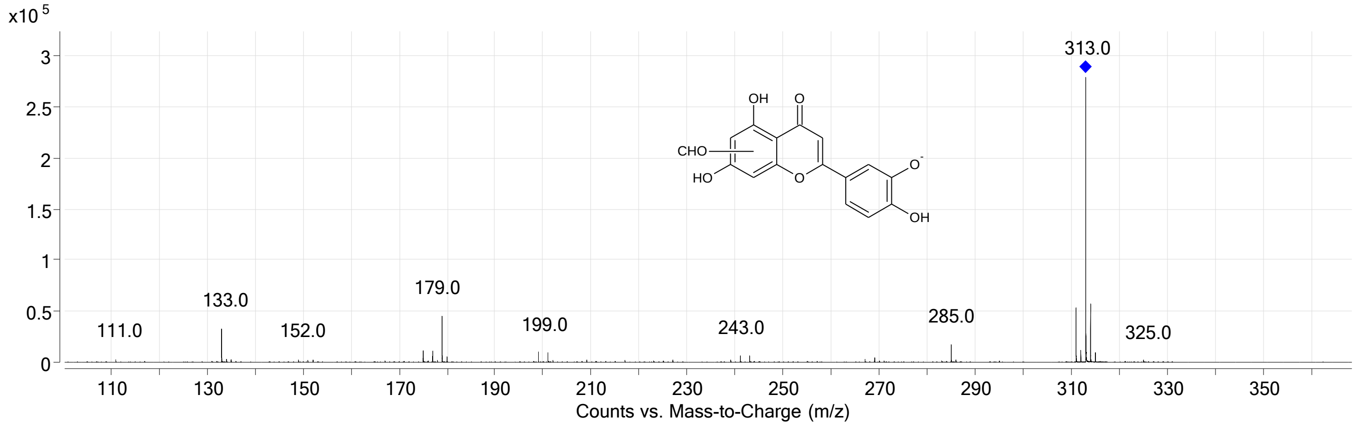

Fig. S15. Mass spectrum of luteolin derivative (Y13).

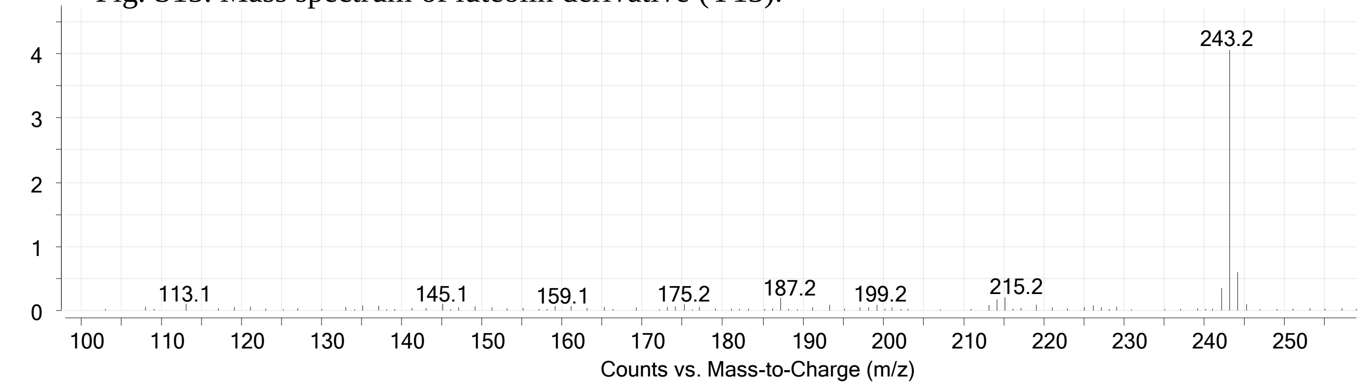

Fig. S16. Mass spectrum of „type C compound" (Y15).

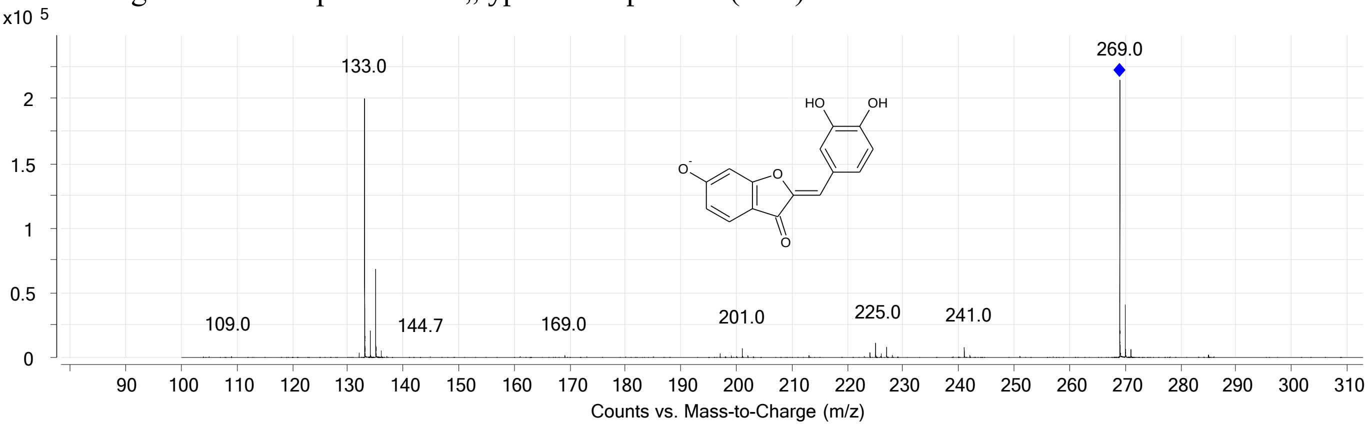

Fig. S17. Mass spectrum of sulfuretin isomer (Y16).

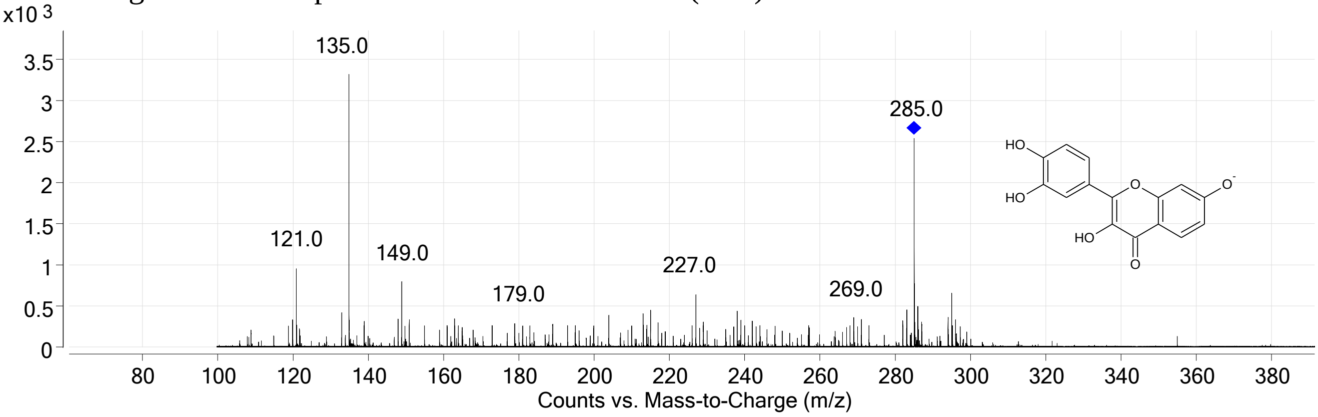

Fig. S18. Mass spectrum of fistein (Y17).

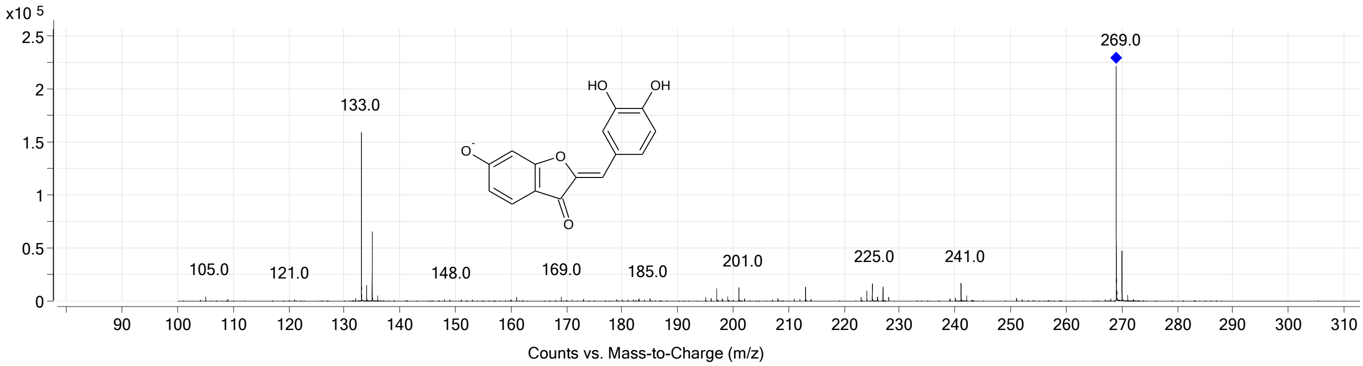

Fig. S19. Mass spectrum of sulfuretin (Y18).

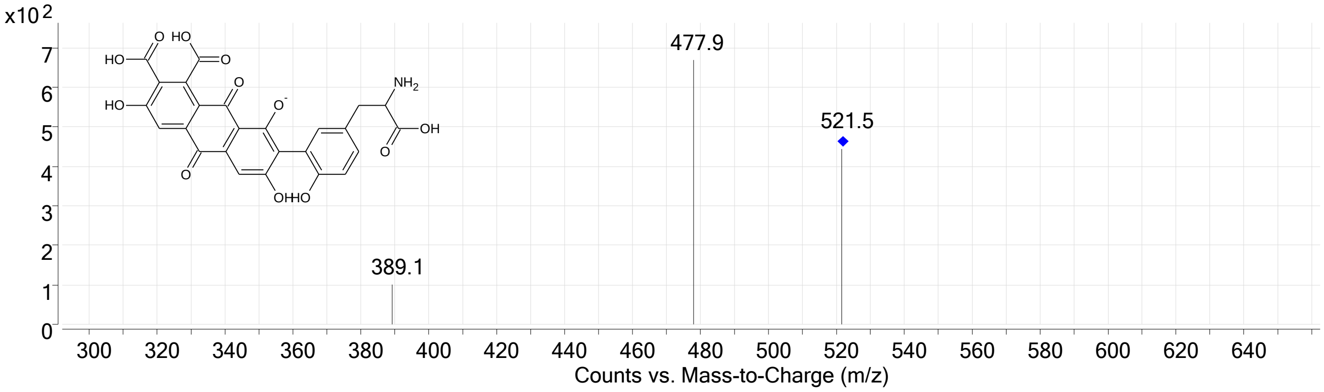

Fig. S20. Mass spectrum of xantholaccaic acid C (R1).

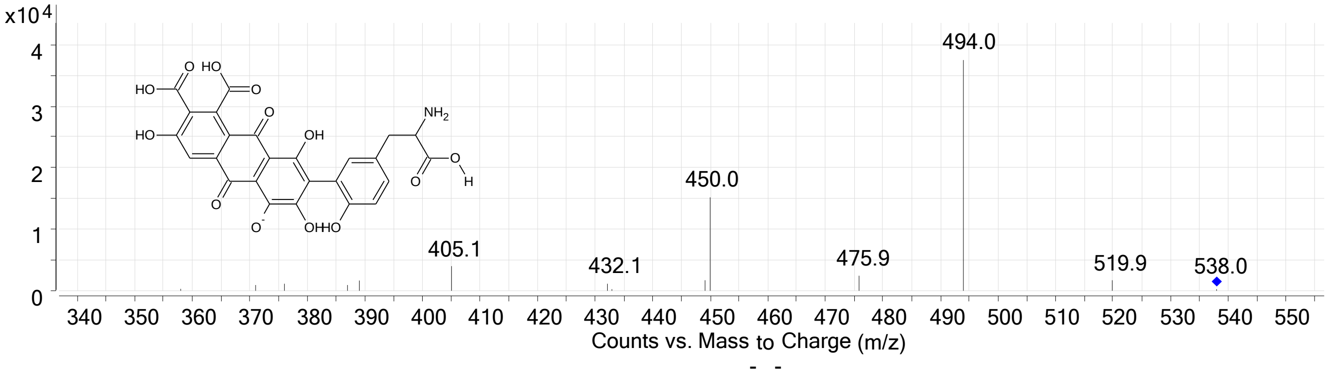

Fig. S21. Mass spectrum of laccaic acid C (R2).

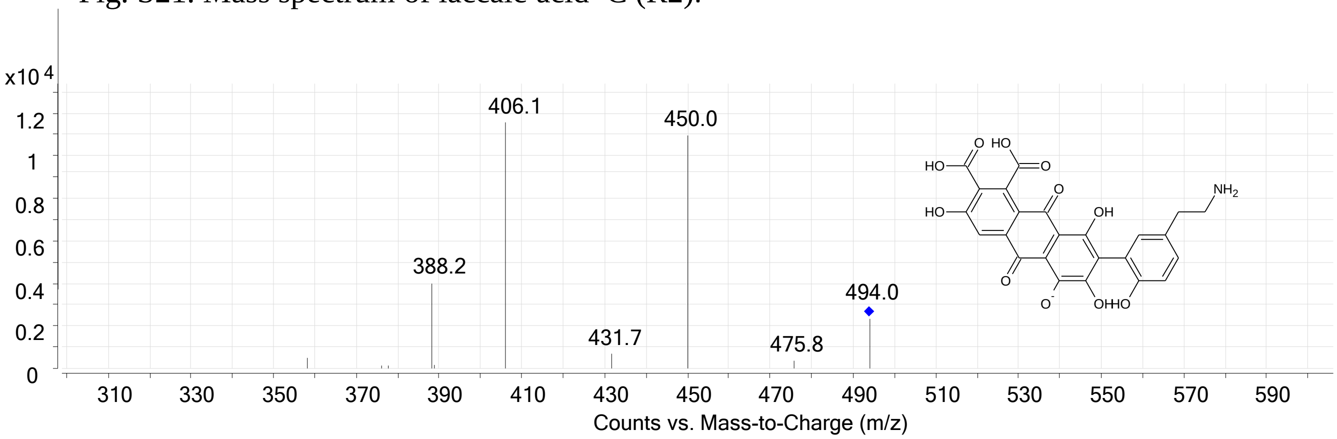

Fig. S22. Mass spectrum of laccaic acid E (R3).

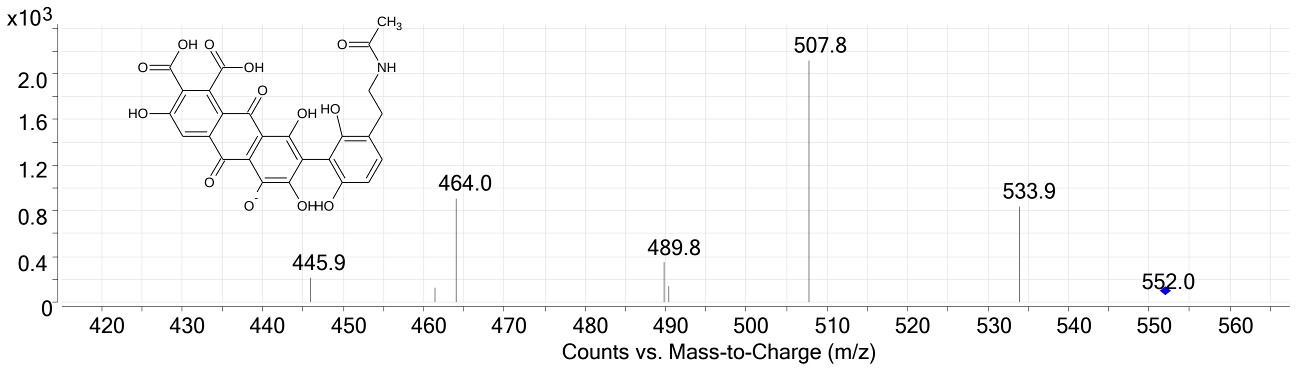

Fig. S23. Mass spectrum of derivative of laccaic acid A (R4).

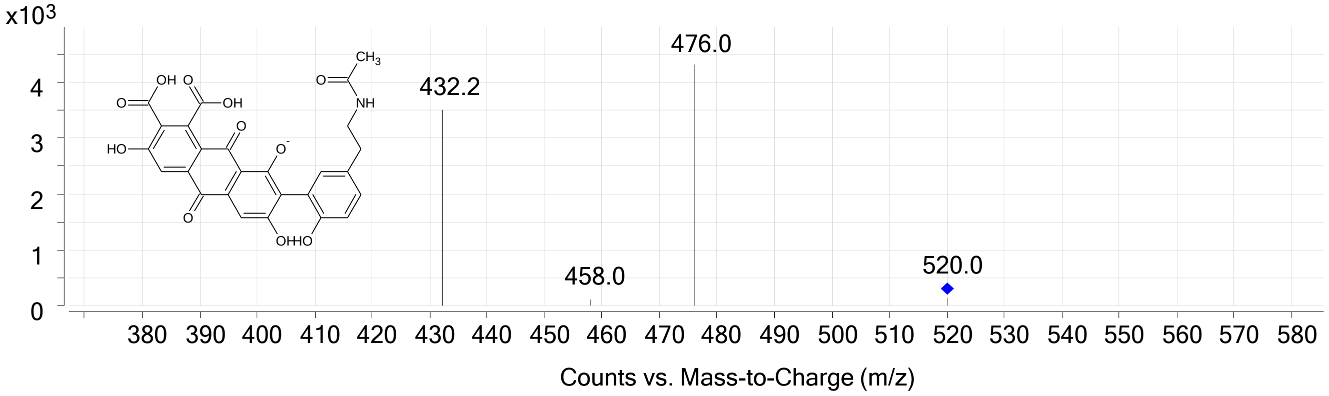

Fig. S24. Mass spectrum of derivative of xantholaccaic acid A (R5).

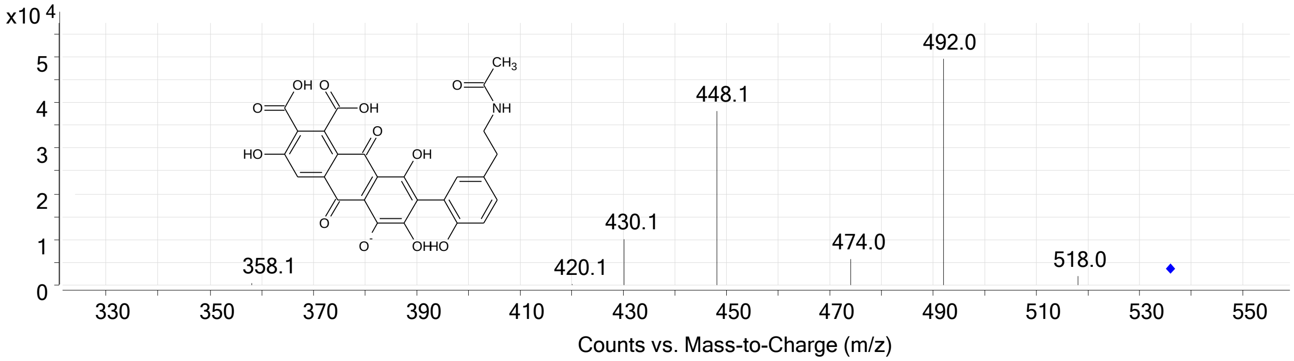

Fig. S25. Mass spectrum of laccaic acid A (R6).

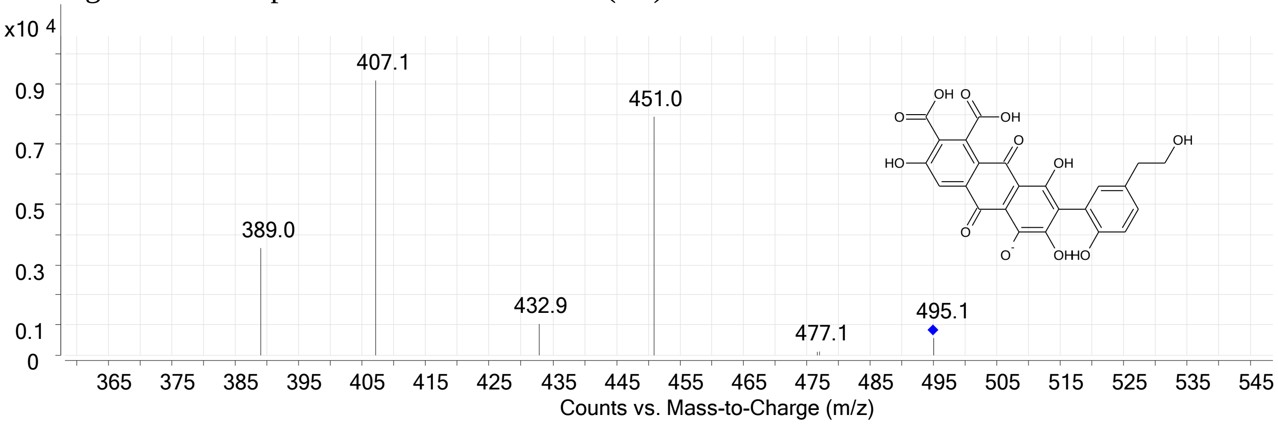

Fig. S26. Mass spectrum of laccaic acid B (R7).

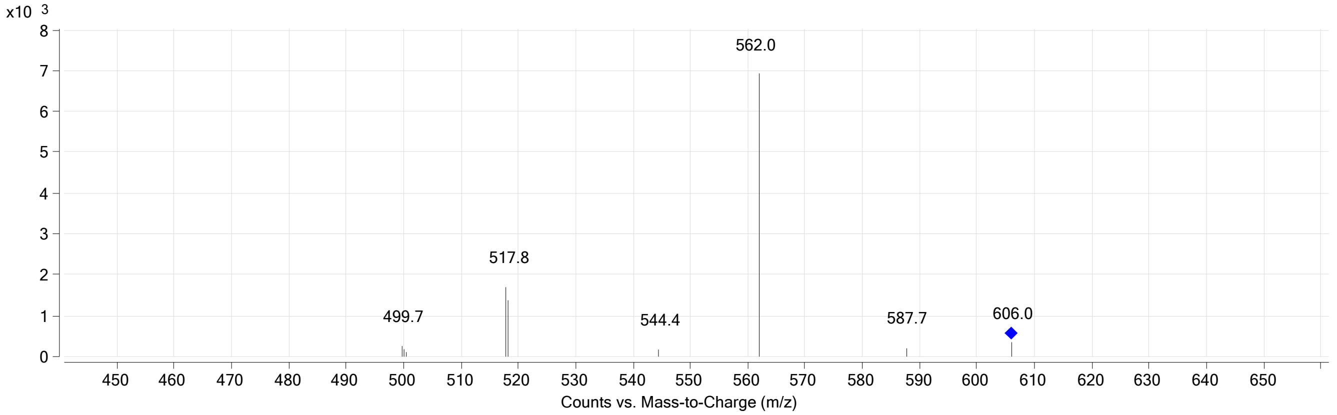

Fig. S27. Mass spectrum of unknown compound (R8).

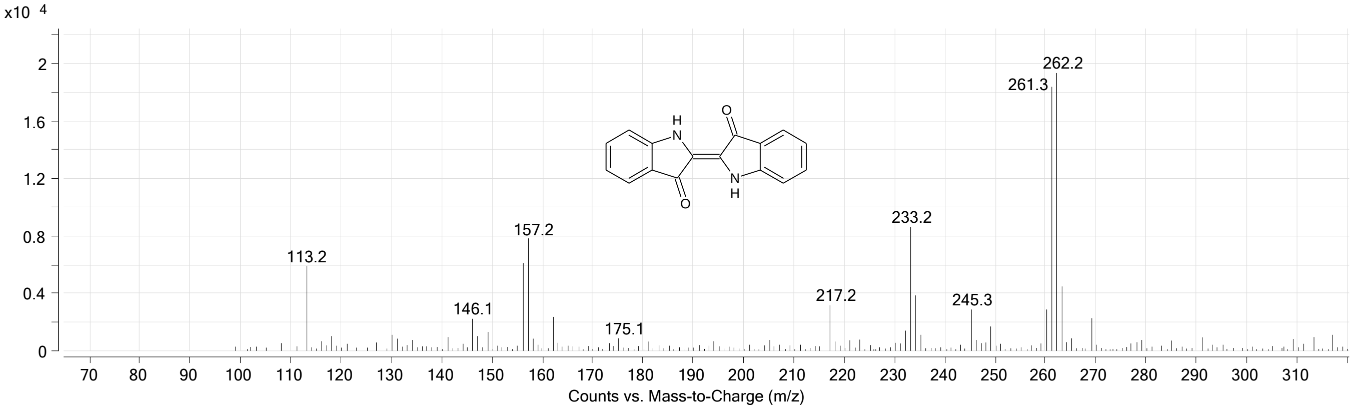

Fig. S28. Mass spectrum of indigotin (B1).

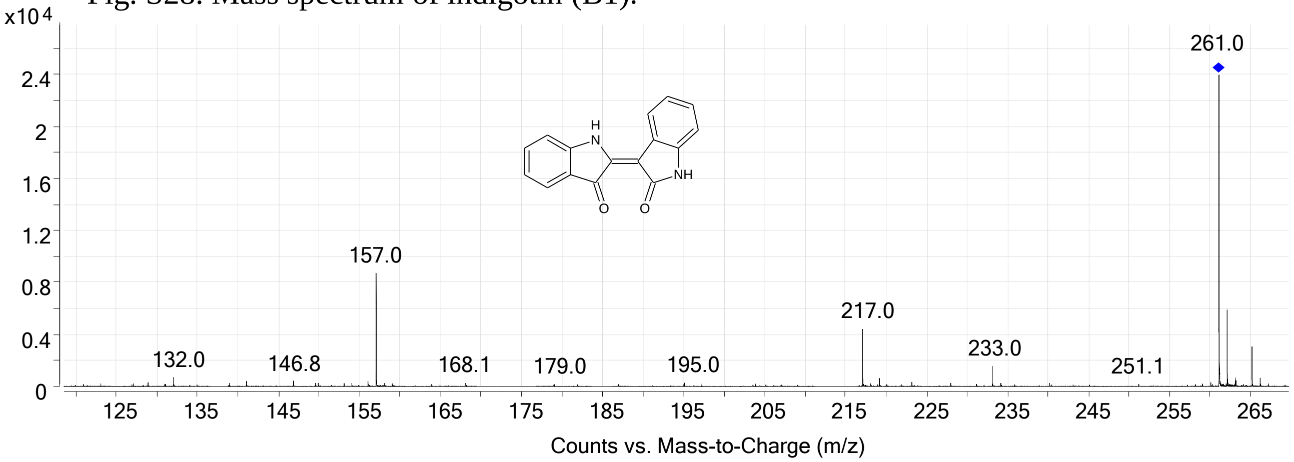

Fig. S29. Mass spectrum of indirubin (B2).

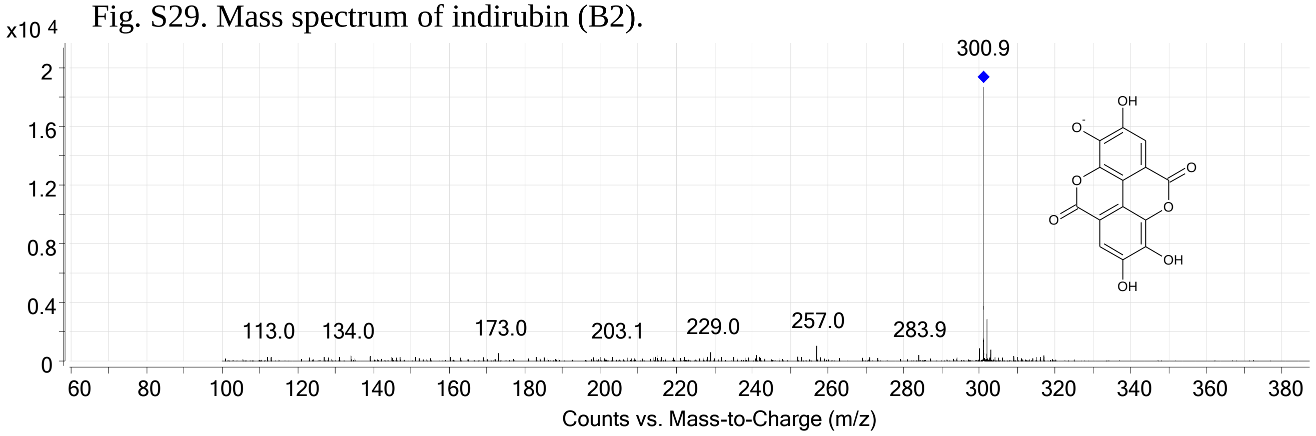

Fig. S30. Mass spectrum of ellagic acid (BR1).

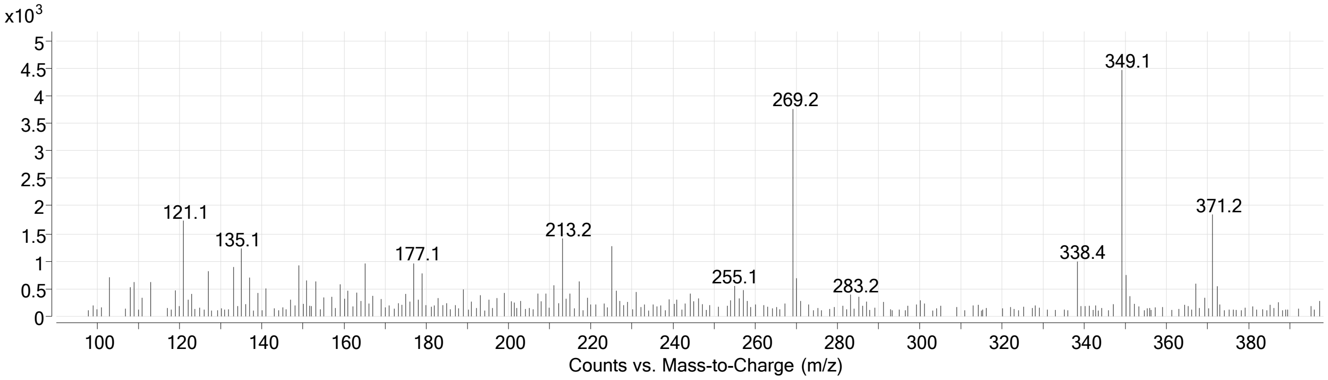

Fig. S31. Mass spectrum of compound (Y14).

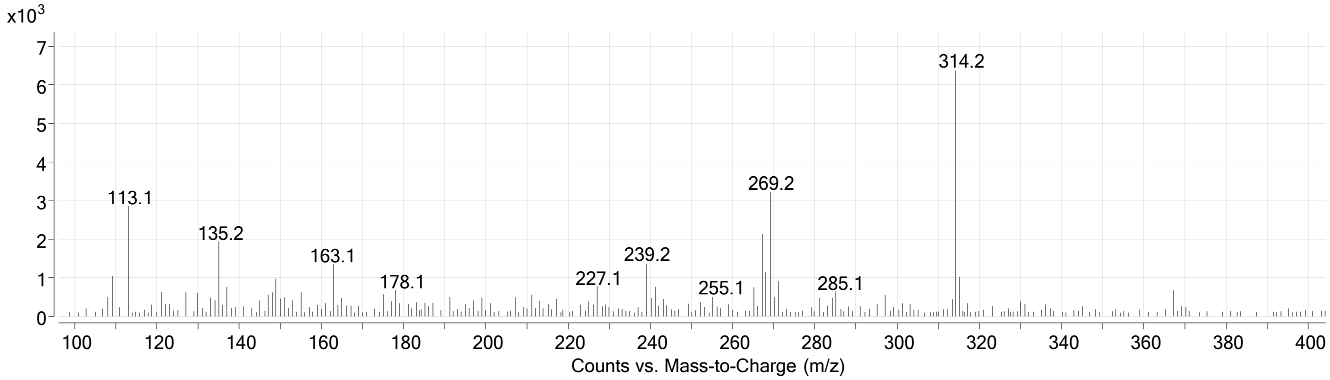

Fig. S32. Mass spectrum of compound (Y19).

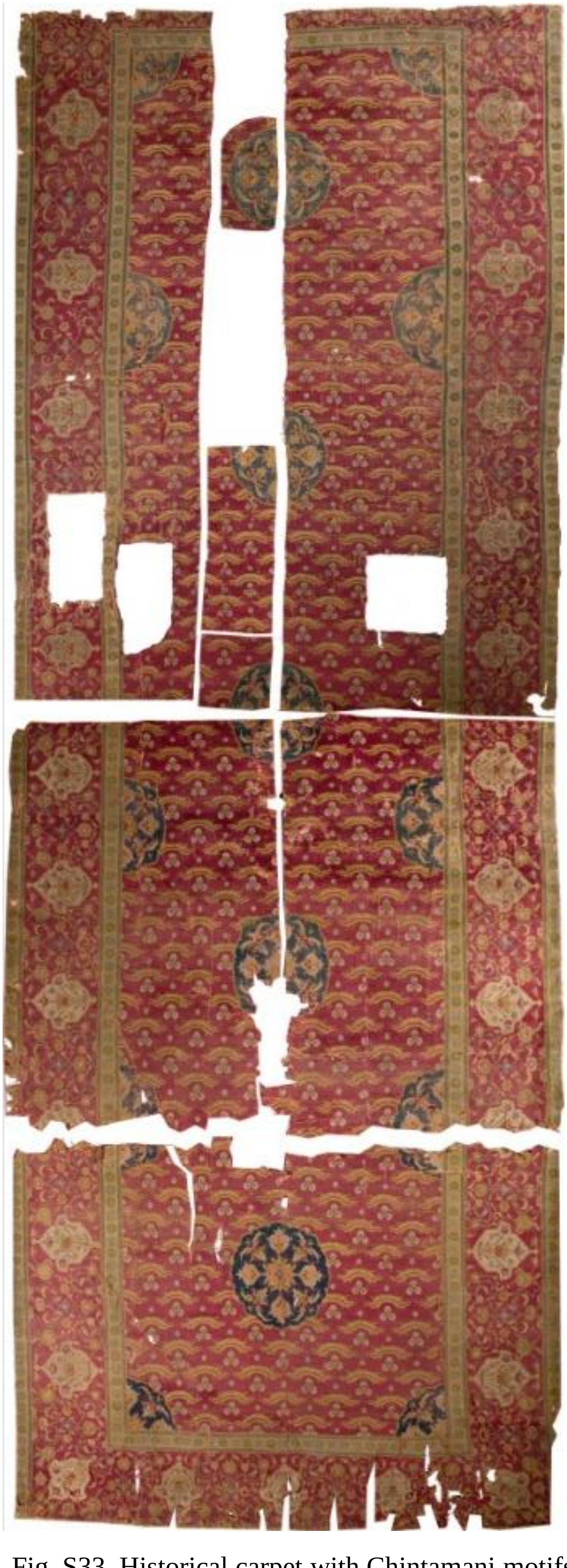

Fig. S33. Historical carpet with Chintamani motifs
(National Museum in Kracow, Poland collection MNK XIX-8950)
